# Prognostic modeling of glioma using epilepsy-related genes highlights PAX3 as a regulator of migration and vorinostat sensitivity

**DOI:** 10.3389/fneur.2025.1665835

**Published:** 2025-10-17

**Authors:** Wei Lin, Haoming Lin, Yaqi Zheng, Jin Wang, Junliang Li, Rui Yang, Zhongfei Zhang, Xiaoping Liu, Xinke Xu

**Affiliations:** ^1^Department of Neurosurgery, Guangzhou Women and Children's Medical Center, Guangzhou Medical University, Guangzhou, China; ^2^Department of Operating Room, Guangzhou Women and Children's Medical Center, Guangzhou Medical University, Guangzhou, China; ^3^School of Public Health, Suzhou Medical College of Soochow University, Suzhou, China; ^4^The Department of Hematology and Oncology, Guangzhou Women and Children's Medical Center, Guangzhou Medical University, Guangzhou, China

**Keywords:** epilepsy, epilepsy-related genes, glioma, prognostic model, PAX3

## Abstract

This study aimed to construct and validate a prognostic model for glioma based on epilepsy-related genes (ERGs) and to investigate the functional role of PAX3 in glioma progression and drug response. Transcriptomic and clinical data from TCGA, GEO, and CGGA databases were used to identify differentially expressed ERGs between glioma patients with and without epilepsy. Univariate Cox regression, LASSO regression, and multivariate Cox analysis were employed to establish a four-gene prognostic model comprising PAX3, RETN, VEPH1, and HTR1A. Patients were stratified into high- and low-risk groups based on the median risk score, which was calculated using gene expression levels and corresponding regression coefficients. The model showed robust prognostic performance, with AUC values exceeding 0.85 in the training set and remaining above 0.73 in internal and external validation cohorts. Kaplan–Meier survival analysis demonstrated significantly longer overall survival in the low-risk group. The risk score was also validated as an independent prognostic factor across multiple datasets. A nomogram integrating clinical features and risk score further improved prediction accuracy, with C-index values up to 0.843 and high calibration concordance. Among the ERGs, PAX3 showed the strongest correlation with the risk score and was overexpressed in glioma, where it promoted proliferation, migration, epithelial–mesenchymal transition, and resistance to vorinostat through regulation of HDAC1/2/3 targets, as confirmed by functional assays showing that PAX3 knockdown suppressed proliferation and migration, while overexpression enhanced these effects. In conclusion, this study developed and validated a four-gene ERG-based prognostic model with high clinical utility and identified PAX3 as a potential therapeutic target that drives glioma cell migration and vorinostat sensitivity.

## 1 Introduction

Glioma, the most prevalent primary tumor of the central nervous system, exhibits characteristics of infiltrative growth, high invasiveness, and a propensity for recurrence (1). According to the 2021 World Health Organization Classification of Tumors of the Central Nervous System, gliomas are categorized as grade IV and are further subdivided into low-grade glioma (LGG) and high-grade glioma (HGG) (2). Patients with LGG typically have a 5-year survival rate of 30% to 70% (3), while those with HGG face a much poorer prognosis, with a median overall survival of ~15 months (4). Despite advancements in conventional therapies like surgical resection, radiotherapy, chemotherapy, and bevacizumab (5), prognosis remains limited for glioma patients, emphasizing the critical need for identifying novel molecular targets to enhance personalized treatment and clinical outcomes. The pursuit of innovative diagnostic approaches, predictive models, and therapeutic targets for glioma stands as a prominent focus of current research endeavors.

Epilepsy, characterized by synchronous abnormal neuronal discharges in the brain, is a common manifestation in various neurological disorders, notably in LGG where seizures may present as the sole clinical symptom associated with glioma. Glioma-associated epilepsy (GRE) affects roughly 30% to 90% of patients (6, 7). While its exact mechanisms are not yet fully understood, GRE is largely believed to result from tumor-intrinsic factors, alterations in the tumor microenvironment, and associated inflammatory processes. GRE typically presents as medically refractory epilepsy, with maximal resection of the tumor and surrounding epileptogenic cortex being pivotal for efficacy, achieving successful seizure control in about 60% to 70% of cases post-surgery. Nevertheless, 30% to 40% of patients continue to experience inadequate seizure control post-surgery (8). Studies indicate that LGG patients presenting solely with epileptic manifestations exhibit higher survival rates compared to those with other clinical symptoms, and individuals with a preoperative epilepsy history generally fare better clinically than those without such a history (9).

Moreover, epilepsy-associated genes may contribute to glioma progression through neuroinflammation, synaptic remodeling, and epigenetic regulation (1). For example, IDH1, NOTCH1, and ERBB2 promote proliferation, angiogenesis, and migration by inducing inflammatory mediators such as IL-1β, TNF-α, VEGF, and TGF-β, which activate NF-κB and Notch signaling. BDNF and SYNGAP1 influence neuron–tumor interactions by modulating synaptic transmission, thereby affecting tumor cell migration. In addition, epigenetic silencing of TP53 through DNA methylation reduces its tumor-suppressor activity, leading to impaired apoptosis and increased proliferation in glioma cells (10).

Hence, elucidating the determinants and pathogenesis of GRE holds significance for enhancing post-surgical seizure control and glioma prognosis. Notably, there have been no reports on the construction of a glioma prognostic model based on epilepsy-related genes (ERGs).

To assess the predictive value of ERGs in glioma prognosis, this study utilized publicly available glioma transcriptome data and clinical information to develop a prognostic prediction model utilizing ERGs. Subsequent validation aimed to explore novel drugs and potential therapeutic targets.

## 2 Materials and methods

### 2.1 Data collection and ERG identification

RNA sequencing data and corresponding clinical information for low-grade glioma (LGG) and glioblastoma (GBM) patients were retrieved from the Cancer Genome Atlas (TCGA) via the UCSC Xena platform (https://xenabrowser.net/). To identify gene expression profiles associated with glioma-related epilepsy, we accessed the Gene Expression Omnibus (GEO) dataset GSE199759. Additionally, two independent glioma cohorts—mRNA-array_301 and mRNAseq_325—were obtained from the Chinese Glioma Genome Atlas (CGGA) database to support external validation.

In this study, ERGs were defined as tumor gene expression patterns significantly associated with the glioma-related epilepsy (GRE) phenotype, rather than genes derived from common epilepsy or non-tumor brain tissue. In two independent discovery cohorts, samples were classified into epilepsy and non-epilepsy groups based on clinical annotations (presence of epilepsy or pre-surgical history of epilepsy). Differential expression analyses were then performed separately, and genes consistently significant in both cohorts were retained as ERGs.

Within the TCGA dataset, only the LGG dataset contained information regarding a patient's epilepsy history. Consequently, we divided the TCGA-LGG dataset into two distinct groups based on seizure history: one consisting of 311 samples with a history of seizures and the other comprising 183 samples without such history. Similarly, we segmented the GSE19985 dataset into two groups, with nine samples displaying a history of seizures and 16 samples without. Utilizing the R package “limma,” we performed differential expression analysis of both datasets, with a focus on identifying ERGs.

#### 2.1.1 Batch effect handling and platform differences

To reduce the impact of platform variation and batch effects, all datasets were standardized before analysis. RNA sequencing data were processed using RSEM or FPKM normalization followed by log_2_ transformation, while the microarray dataset (mRNA_array_301) was quantile normalized and log_2_ transformed to ensure cross-platform comparability (Supplementary Table S1).

### 2.2 Prognostic model construction and validation based on ERGs

Following the removal of samples lacking survival information, we utilized the “care” package to randomly partition the TCGA-LGG and GBM dataset into two sets in a 6:4 ratio based on survival status, designating them as the training and internal test sets, respectively. The other two datasets obtained from the CGGA database served as independent validation sets.

Survival-associated epilepsy-related genes (ERGs) were first identified using univariate Cox regression analysis. Overall survival was the outcome of interest. Each ERG was first assessed using univariate Cox regression (Wald test), with multiple testing corrected by the Benjamini–Hochberg method (FDR < 0.05). Twenty-five genes were identified as significant. To address multicollinearity and reduce overfitting, these genes were then subjected to LASSO-penalized Cox regression with 10-fold cross-validation, using the λ_1-*se*_ criterion to favor a simpler and more generalizable model. Four genes with non-zero coefficients were retained and entered into multivariable modeling. Forward stepwise selection based on the likelihood ratio test and Akaike information criterion (AIC) yielded a final four-gene signature (PAX3, RETN, VEPH1, and HTR1A). All four remained statistically significant and directionally consistent with the univariate results, and Schoenfeld residuals confirmed no violation of the proportional hazards assumption.

The risk score for each patient was calculated based on the expression levels of ERGs (*Expi*) and their corresponding Cox regression coefficients (*coefi*), using the formula: Risk score = ∑ (Expi × coefi). LASSO regression was implemented with the “glmnet” package (11). Based on the median risk score, patients in both the training and testing cohorts were stratified into high-risk and low-risk groups.

Univariate and multivariate Cox regression analyses were performed using the “survival” and “survminer” packages to assess whether the risk score independently predicted survival, and Kaplan–Meier survival curves were generated accordingly.

To evaluate the predictive performance of the model, time-dependent receiver operating characteristic (ROC) curve analysis was conducted using the “survivalROC” package (12) and the “timeROC” package (13). The area under the ROC curve (AUC) was calculated to quantify the model's discriminative power at different time points.

Additionally, a nomogram integrating the risk score and clinical features was constructed using the “rms” package. The model's predictive accuracy was assessed by the concordance index (C-index) and calibration plots. External validation of the ERG-based prognostic model was performed using glioma cases from the CGGA dataset to confirm its robustness.

### 2.3 Correlation analysis

To investigate the biological and clinical relevance of the ERG-based risk score, we conducted correlation analyses between the risk score and multiple factors, including oncogene expression, anti-cancer drug sensitivity, immune checkpoint markers, and immune cell infiltration. Spearman correlation analysis was performed using the “psych” R package.

Oncogene expression data were retrieved from the ONGene database (http://www.ongene.bioinfo-minzhao.org) (14). Drug sensitivity profiles were obtained from the Genomics of Drug Sensitivity in Cancer (GDSC) database (15). Drug response prediction for individual glioma samples was performed using the “oncoPredict” package based on the GDSC V2.0 reference set (16). Predicted sensitivity scores were inferred from IC50 values derived from gene expression data, where higher scores corresponded to reduced drug sensitivity, due to a positive correlation with IC50.

This multi-level correlation analysis aimed to uncover potential links between the ERG signature and oncogenic pathways, therapeutic vulnerabilities, and the tumor immune microenvironment.

### 2.4 Differential expression and enrichment analysis

Differentially expressed genes (DEGs) between the high- and low-risk groups were identified using the “limma” package in R, applying stringent thresholds of |log_2_(fold change)| > 1 and P < 0.05. To investigate the biological functions and pathways associated with these DEGs, functional enrichment analysis was performed using the DAVID online tool (https://davidbioinformatics.nih.gov/summary.jsp).

To further explore the biological processes and signaling pathways associated with the ERG-based risk score, we performed gene set enrichment analysis (GSEA). The analysis focused on genes ranked by their correlation with the risk score and was conducted using curated gene sets from the Molecular Signatures Database (MSigDB), including Gene Ontology (GO) biological processes and Kyoto Encyclopedia of Genes and Genomes (KEGG) pathways. Gene set collections were obtained from the MSigDB portal (https://www.gsea-msigdb.org/gsea/downloads.jsp).

### 2.5 Plasmid construction and transfection

To silence the expression of *PAX3*, we designed specific shRNA sequences using the BLOCK-iT™ RNAi Designer tool (https://rnaidesigner.thermofisher.com/rnaiexpress). After careful selection and testing of various candidate shRNAs, we identified the sequence 5'-GGGCATGTTCAGCTGGGAAAT-3' as the most effective for targeting *PAX3* for knockdown. This selected sequence was then cloned into the pGreen vector. A scrambled, non-specific shRNA (shNC), sequence: 5-CAACAAGATGAAGAGCACCAA-3 was cloned into the pGreen vector and used as a negative control to validate the specificity of gene silencing. For overexpression studies, we amplified the full coding sequence of *PAX3* and inserted it into the pCDH vector. This approach allowed us to investigate the effects of *PAX3* overexpression on cellular processes.

### 2.6 Cell culture and transfection

Human glioblastoma (GBM) cell lines U87 and U251 were obtained from the American Type Culture Collection (ATCC) and maintained in Dulbecco's Modified Eagle Medium (DMEM) supplemented with 10% fetal bovine serum (FBS). Cells were cultured at 37 °C in a humidified incubator with 5% CO_2_.

For transfection experiments, cells were seeded into 6-well plates and allowed to adhere for 24 h. Transfection was performed using Lipofectamine 6,000 reagent (Beyotime, China) following the manufacturer's instructions. Each well was transfected with 2.5 μg of either shPAX3 plasmid or a PAX3 overexpression construct.

### 2.7 Cell proliferation assays

To assess cell proliferation, we performed both CCK-8 and EdU incorporation assays.

For the CCK-8 assay, cells were seeded into 96-well plates at a density of 5 × 103 cells per well. After 24 h of incubation, 10 μl of Cell Counting Kit-8 (CCK-8; Beyotime, China) reagent was added to each well, followed by a 2-h incubation at 37 °C. Absorbance was measured at 450 nm using a microplate spectrophotometer. Each condition was tested in triplicate to ensure reproducibility.

For the EdU assay, cells were plated in 6-well plates and incubated with 10 μM 5-ethynyl-2'-deoxyuridine (EdU) for 2 h. Following incubation, cells were fixed with 4% paraformaldehyde and permeabilized with 0.3% Triton X-100 in PBS. EdU staining was performed using a commercial kit (Beyotime Institute of Biotechnology, China) according to the manufacturer's protocol. Images were captured within 24 h using an inverted fluorescence microscope, and EdU-positive cells were quantified using NIH ImageJ software (version 1.8.0), providing a measure of proliferative activity.

### 2.8 Cell migration assays

To perform a wound healing assay, cells were cultured in 6-well plates waiting for full confluency. Subsequently, a uniform scratch was created across the cell monolayer using a sterile pipette tip, under condition of serum-free medium.

For the wound healing assay, GBM cells were seeded into 6-well plates and cultured until a confluent monolayer was formed. A straight scratch was then introduced across the cell monolayer using a sterile 200-μl pipette tip. To minimize cell proliferation and focus on migratory behavior, the medium was replaced with serum-free DMEM. Time-lapse images were captured at designated intervals using an inverted microscope to track the closure of the scratch and wound closure was quantified by measuring the remaining gap area using ImageJ software.

To conduct the Transwell migration assay, cells were seeded into the upper chamber of the Transwell inserts (Corning, Inc., USA) with a serum-free medium (1 ml), while the lower chamber contained 2 ml of complete medium. After incubation for 24 h at 37 °C, non-migrated cells on the upper surface of the membrane were removed, and migrated cells on the lower surface were fixed (4% paraformaldehyde), stained with crystal violet (0.5% solution), and imaged. The extent of cell migration was quantified by analyzing the stained cells with ImageJ software.

### 2.9 Western blotting

For western blotting, cellular proteins were extracted using RIPA lysis buffer (Beyotime, China), and their concentrations were determined with the Enhanced BCA Protein Assay Kit (Beyotime, China). Equal amounts of protein (30 μg per sample) were resolved by SDS-PAGE and transferred onto polyvinylidene fluoride (PVDF) membranes (MilliporeSigma, Billerica, MA, USA). After blocking with 5% bovine serum albumin, the PVDF membranes were incubated overnight at 4 °C with primary antibodies (diluted 1:1,000) against GAPDH (Cell Signaling Technology, USA), N-cadherin (CDH2, ProteinTech, USA), and E-cadherin (CDH1, ProteinTech, USA).

After primary antibody incubation, membranes were probed with horseradish peroxidase-conjugated secondary antibodies. The protein bands were visualized utilizing enhanced chemiluminescence reagent (GeneTools GBox system, Syngene). Subsequently, the band intensity was quantified using ImageJ software (National Institutes of Health), with GAPDH serving as a reference for normalization.

### 2.10 Analysis of vorinostat sensitivity

Cell viability was measured using the CCK-8 assay after treatment with vorinostat across a concentration gradient of 0, 0.5, 1, 2, 4, 8, 16, and 32 μM. Absorbance at 450 nm was measured to quantify viability. Utilizing GraphPad software, the IC50 values were determined, representing the vorinostat concentration required for 50% cell inhibition. Comparative analysis between the shNC and shPAX3 groups provided insights into the impact of PAX3 knockdown on vorinostat sensitivity (17).

### 2.11 Statistics analysis

All statistical analyses were performed using GraphPad Prism version 8.3.0 (GraphPad Software, LLC). Data are presented as mean ± standard deviation (SD). Comparisons between two groups were evaluated using Student's *t*-test, while one-way ANOVA was applied for analyses involving more than two groups. A *P* value < 0.05 was considered statistically significant.

## 3 Results

### 3.1 Data collection and development of prognostic models

To construct and validate prognostic models, we collected data from three independent cohorts of lower-grade glioma and glioblastoma (LGG-GBM) patients, integrating both genomic and clinical information derived from the Cancer Genome Atlas (TCGA) and the Gene Expression Omnibus (GEO), with emphasis on the GSE39582 dataset. The clinical characteristics and demographic information of patients included in the training, internal testing, and external validation cohorts are detailed in Supplementary Tables S2, S3. From the TCGA-LGGGBM dataset, which originally contained 659 samples, cases lacking essential clinical data were excluded. The remaining eligible cases were randomly split into a training set (*n* = 496; 60%) and an internal testing set (*n* = 263; 40%). Comparative analysis confirmed that the key clinicopathological variables were well balanced across the training, internal testing, and full TCGA-LGGGBM cohort, with no statistically significant differences observed (*P* > 0.05; Supplementary Table S2).

Through comparative analysis of gene expression patterns among patients with and without a seizure history, we identified several down-regulated genes within both the GEO databases (38 genes) and the TCGA-LGGGBM dataset (3,695 genes), along with 188 up-regulated genes in the GEO databases and 3,904 in the TCGA-LGGGBM dataset. These findings adhered to predefined criteria of |log_2_ fold change| > 1 and *P-*value < 0.05 (Supplementary Figures S1A, B). Subsequently, by intersecting these datasets, we pinpointed 34 common prognostic-related genes in glioma (Supplementary Figures S1C, D).

**Figure 1:**
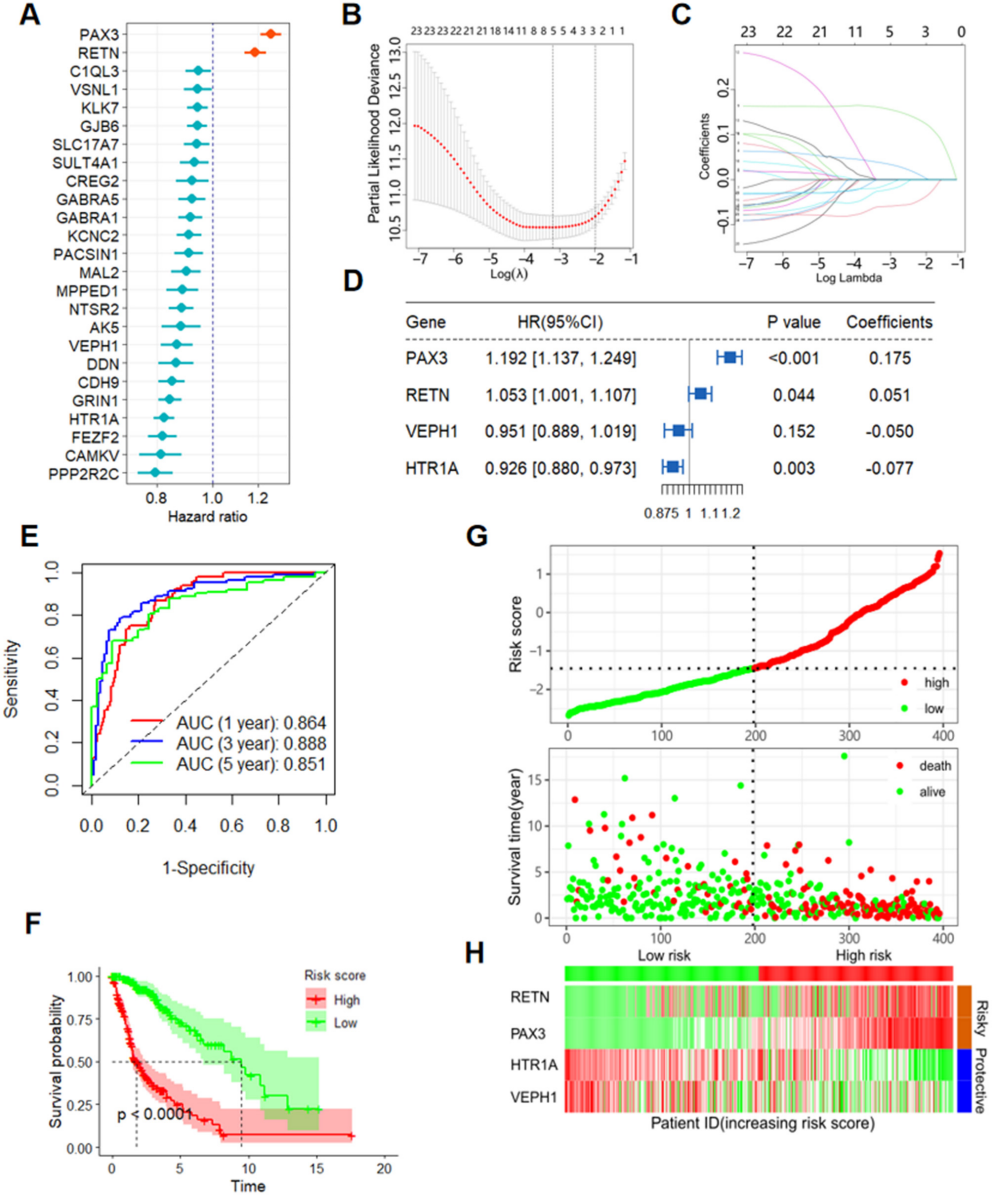
Development of an ERG-based prognostic model for glioma. **(A)** OS-related ERGs identified in glioma patients by univariate Cox regression analysis. **(B, C)** Four ERGs for OS identified by LASSO-penalized Cox analysis. **(D)** Forest plot of the results of multivariate Cox regression analysis of four ERGs. **(E)** ROC curves depicting the predictive performance for 5-year OS in the training set. **(F)** Kaplan–Meier curve illustrating OS in the training group. **(G)** Distribution of risk scores and survival status within the training group. **(H)** Heatmap visualizing the expression of four ERGs within the training group. ERGs, epilepsy-related genes; LASSO, least absolute shrinkage and selection operator; OS, overall survival; ROC, receiver operating characteristic.

Utilizing univariate Cox regression analysis on the training set, we identified 25 ERGs related to prognosis (Figure 1A). Subsequently, employing LASSO-penalized Cox analysis, we further narrowed these down to four ERGs for multivariate analysis (Figures 1B, C). We constructed a multivariate Cox proportional hazard model through step-wise implementation of the likelihood-ratio forward method, yielding a risk model for assessing the prognostic risk of glioma patients. The formula for the risk score calculation was as follows: Risk score = (0.175 × PAX3 Exp) + (0.051 × RETN Exp) + (−0.05 × VEPH1 Exp) + (−0.077 × HTR1A Exp; Figure 1D).

Receiver operating characteristic (ROC) curve analysis demonstrated strong prognostic performance of the risk score in predicting overall survival (OS) among glioma patients, yielding area under the curve (AUC) values exceeding 0.851 for 1- through 5-year survival intervals (Figure 1E). Kaplan–Meier curves confirmed significantly longer OS in the low-risk group compared to the high-risk group (Figure 1F). Patients in the training cohort were categorized into high- and low-risk groups according to the median risk score, allowing visualization of the distribution of risk scores alongside survival outcomes and durations (Figure 1G). Additionally, a heatmap illustrated the relative expression patterns of the four ERGs across the cohort, offering further insight into molecular differences between risk groups (Figure 1H).

### 3.2 Evaluation of prognostic performance of the ERG-based signature in training and validation cohorts

**Figure 2:**
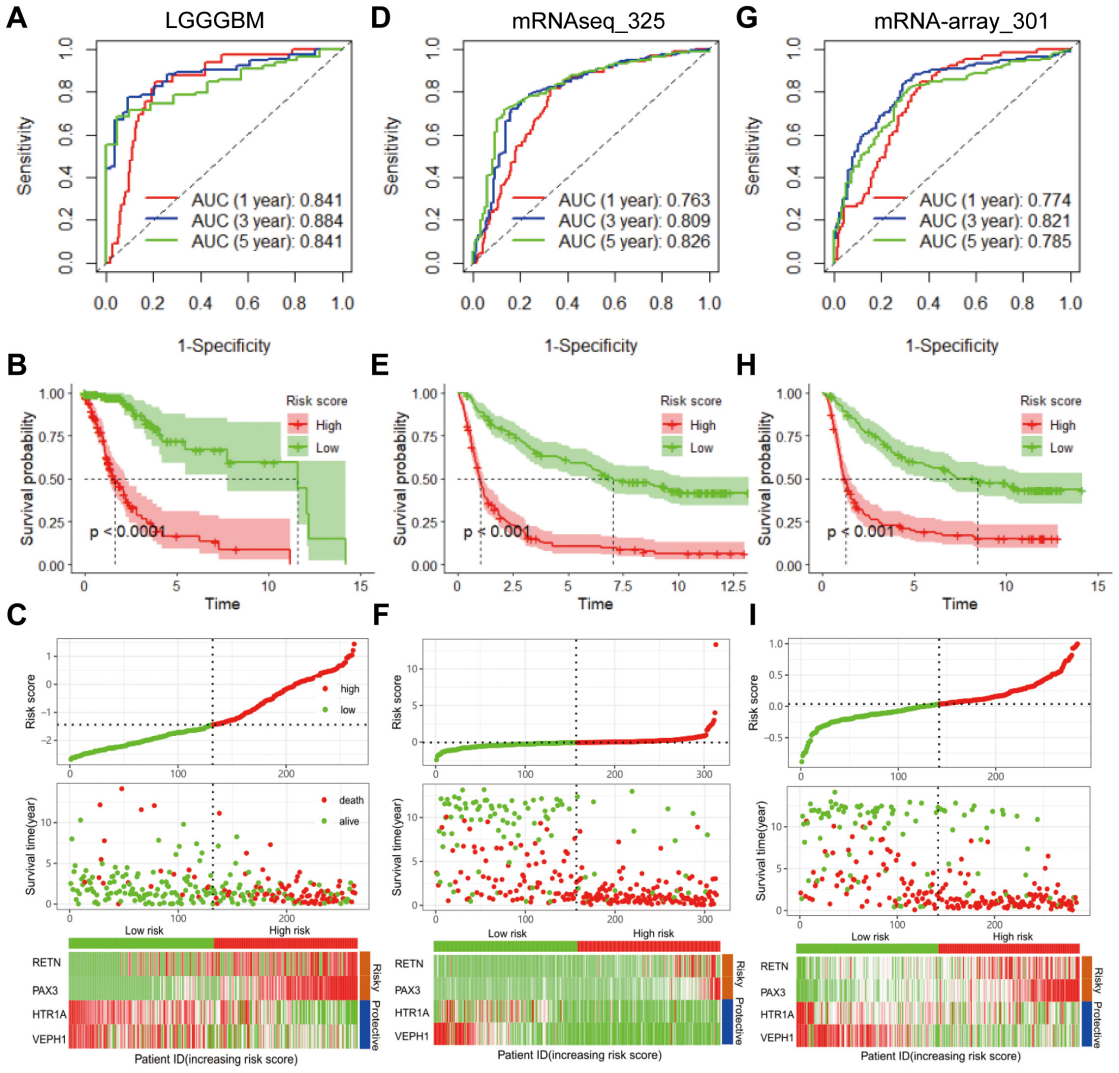
Validation of the prognostic model utilizing the four ERGs constructed from the training dataset. **(A)** ROC curves for OS in TCGA-LGGGBM datasets. **(B)** Kaplan–Meier curve of OS in TCGA-LGGGBM datasets. **(C)** Risk score distribution, survival status, and expression levels of four ERGs in TCGA-LGGGBM datasets. For the validation sets, **(D)** showcases ROC curves for OS in mRNAseq_325 datasets, while **(E)** presents the Kaplan–Meier curve of OS in mRNAseq_325 datasets. **(F)** further details the risk score distribution, survival status, and expression levels of the 4 ERGs in mRNAseq_325 datasets. Lastly, **(G)** exhibits ROC curves for OS in mRNA-array_301 datasets, followed by **(H)**, which illustrates the Kaplan–Meier curve of OS in mRNA-array_301 datasets. Finally, **(I)** depicts the risk score distribution, survival status, and expression levels of the 4 ERGs in mRNA-array_301 datasets. “ERGs” refer to epilepsy-related genes, while “ROC” denotes the receiver operating curve. ERGs, epilepsy-related genes; GBM, glioblastoma; LGG, lower grade glioma; OS, overall survival; ROC: receiver operating characteristic; TCGA-LGGGBM, The Cancer Genome Atlas Merged Cohort of LGG and GBM.

To assess the prognostic relevance of the ERG model, patients from both the internal testing cohort and the complete TCGA-LGGGBM dataset were stratified into high- and low-risk groups according to the median risk score. In both cohorts, individuals within the low-risk group demonstrated markedly improved overall survival (OS) relative to those in the high-risk group (*P* < 0.0001), with the corresponding AUC values surpassing 0.733 and 0.841, respectively (Supplementary Figures S2A, B, Figures 2A, B). Mortality analysis showed a clear association between elevated risk scores and increased death rates, as illustrated by the distribution of survival status across risk groups (Supplementary Figure S2C, Figure 2C). A heatmap further revealed distinct expression profiles of the 20 seizure-associated genes, distinguishing low-risk from high-risk patients (Supplementary Figure S2C, Figure 2C). To confirm the robustness of this ERG signature, external validation was performed using two independent CGGA datasets. In the mRNAseq_325 dataset, the model achieved an AUC above 0.763 (Figures 2D–F), while in the mRNA-array_301 dataset, it exceeded 0.774 (Figures 2G–I), collectively supporting the predictive accuracy and generalizability of the four-gene signature.

### 3.3 ERGs risk score as an independent and robust predictive indicator

The ERGs risk score emerges as a standalone and robust predictor, as illustrated in Supplementary Table S4. It exhibits correlations with various clinical–pathological features within the TCGA-LGGGBM dataset, including age, Karnofsky Performance Status score, *IDH* mutation, and grade. Similarly, it also demonstrates associations with grade, age, and *IDH* mutation in the mRNA_array_301 dataset (Supplementary Table S5), and with grade, *IDH* mutation, and MGMTp methylation status in the mRNAseq_325 dataset (Supplementary Table S6).

**Figure 3:**
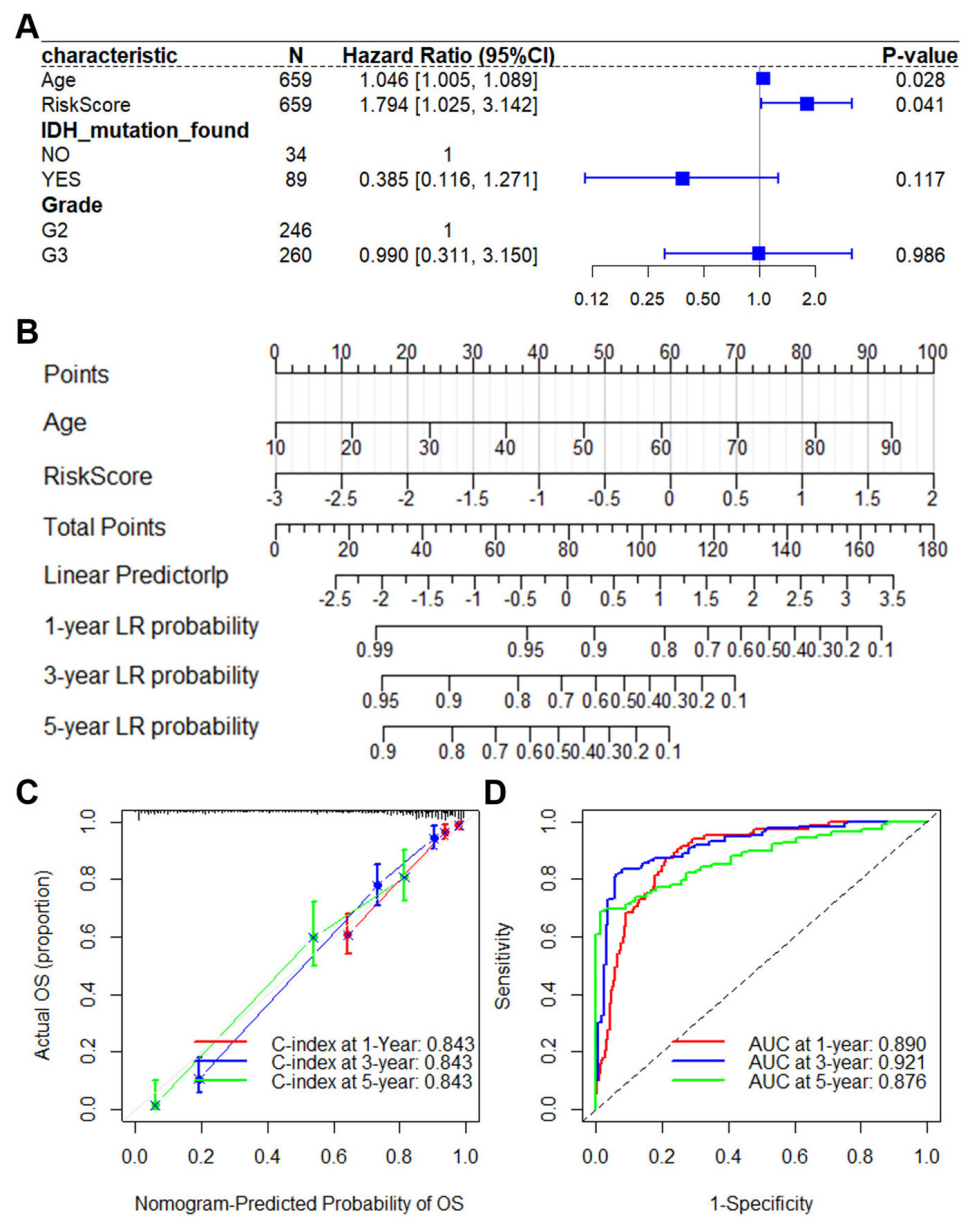
Significance of the ERG risk score as an independent and robust predictive indicator within TCGA-LGGGBM dataset. **(A)** Results of multivariate Cox regression analyses, showcasing the influence of the risk score alongside clinical–pathological features identified from univariate Cox analysis on OS in the TCGA-LGGGBM dataset. **(B)** Nomogram incorporating the 4-SRG risk score and age, derived from the TCGA-LGGGBM dataset. By summing the points from these variables, one can determine the total points, projecting onto the bottom scales to ascertain the probability of 1-, 3-, and 5-year OS. **(C)** Calibration plot for 1-/3-/5-year intervals, validating the accuracy of the prognostic nomogram. **(D)** Kaplan–Meier curve illustrating OS for the score calculated from the nomogram. ERGs, epilepsy-related genes; GBM, glioblastoma; LGG, lower grade glioma; OS, overall survival; TCGA-LGGGBM, The Cancer Genome Atlas Merged Cohort of LGG and GBM.

To assess whether the risk score serves as an independent prognostic factor in glioma, we performed univariate Cox regression analysis for each clinical and pathological variable (Supplementary Figure S3). As illustrated in Figure 3A, the risk score retained strong prognostic significance in the TCGA-LGGGBM cohort even after adjusting for potential confounders in the multivariate model. This independent predictive value was further corroborated in two external datasets, mRNA-array_301 (Supplementary Figure S4A) and mRNAseq_325 (Supplementary Figure S5A), reinforcing the model's applicability across diverse glioma populations.

To ensure the model's robustness and applicability, we developed a prognostic nomogram for predicting OS in glioma patients. This nomogram was built using data from the TCGA-LGGGBM dataset (Figure 3B) as well as other CGGA datasets (Supplementary Figures S4B, S5B). It amalgamates major clinical–pathological features and risk scores. Internal validation through calibration plots and computation of the bootstrap C-index in TCGA-LGGGBM (C-index = 0.843, Figure 3C), mRNA-array_301 (C-index = 0.755, Supplementary Figure S4C), and mRNAseq_325 (C-index = 0.688, Supplementary Figure S5C) reaffirms its reliability.

The ROC curve confirms that the nomogram-derived score effectively predicts OS, with AUC values of 0.921, 0.876, and 0.837 at 3-year intervals in TCGA-LGGGBM (Figure 3D), mRNA-array_301 (Supplementary Figure S4D), and mRNAseq_325 (Supplementary Figure S5D), respectively. These findings underscore the robust predictive capability of the prognostic model based on the four EGRs.

### 3.4 Intriguing correlations of the ERGs risk score with immune cell infiltration and the expression of immune checkpoints

**Figure 4:**
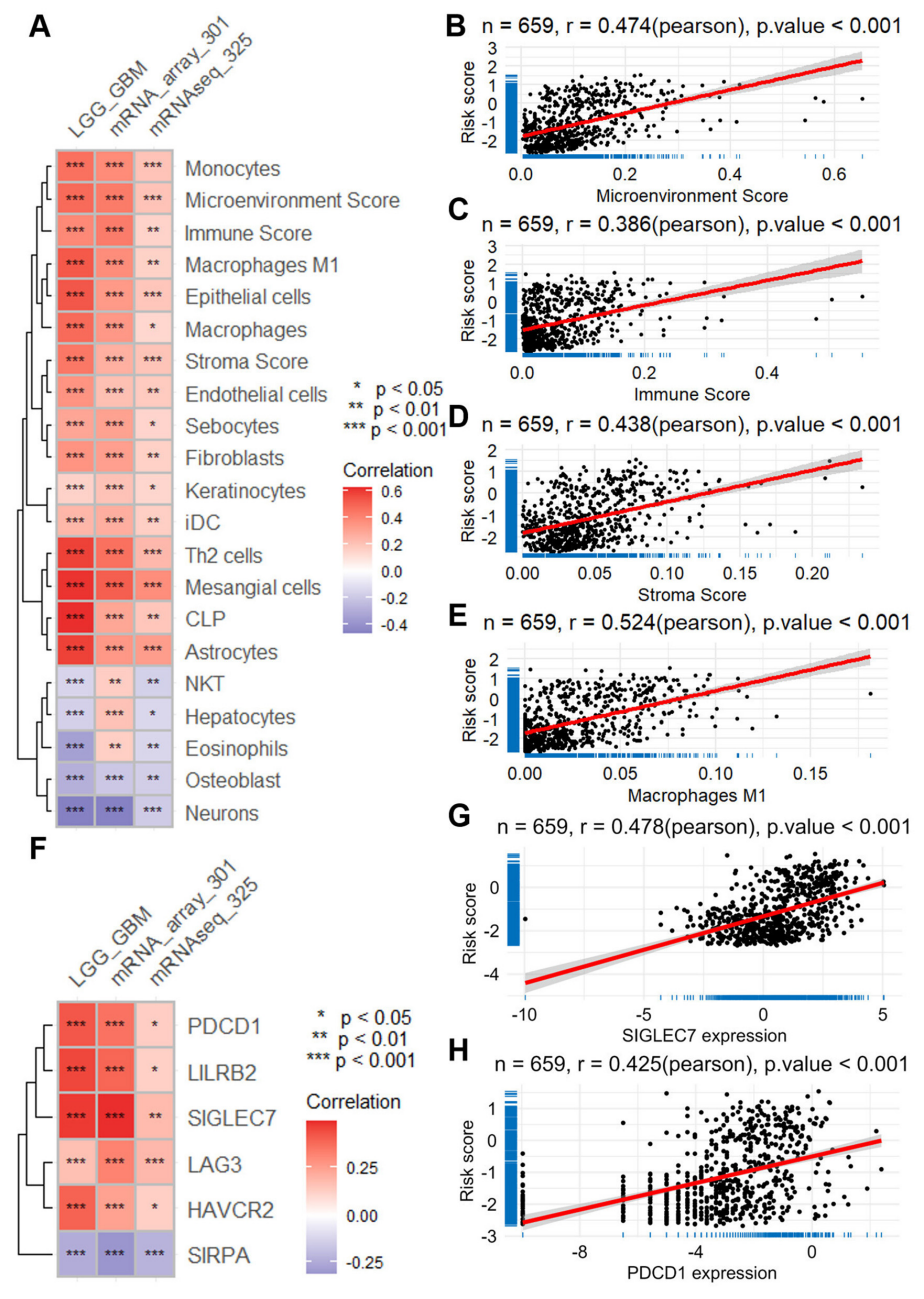
Relationship between ERG risk score and immune cell infiltration, as well as the expression of immune checkpoint genes. **(A)** Heatmap visualizing the correlation between risk score and immune cell infiltration, calculated with the XCELL algorithm using data across the TCGA-LGGGBM dataset and two CGGA datasets. Scatter plots illustrate the correlation between the risk score and the scores of microenvironment **(B)**, immune **(C)**, and stroma **(D)**, as well as the correlation between the risk score and the infiltration of phagocytes **(E)**. **(F)** Heatmap presenting the correlation between the risk score and the expression of immune checkpoint genes across the same datasets. Scatter plots demonstrate the correlation between the risk score and the expression of specific immune checkpoint genes, such as *SIGLEC7* **(G)** and *PDCD1* **(H)**. CGGA, Chinese Glioma Genome Atlas; ERG, epilepsy-related gene; GBM, glioblastoma; LGG, lower grade glioma; TCGA-LGGGBM, The Cancer Genome Atlas Merged Cohort of LGG and GBM.

To quantify immune cell infiltration levels, we applied the “XCELL” algorithm, which performs single-sample gene set enrichment analysis (ssGSEA) to estimate the relative abundance of various immune cell types. Our correlation analyses unveiled significant associations between the risk score and the infiltration of multiple immune cell types across the TCGA-LGGGBM dataset and two additional CGGA datasets (Figure 4A).

Notably, the risk score demonstrated a moderate positive correlation with the tumor microenvironment components, including microenvironment score (*r* = 0.470; TCGA-LGGGBM, Figure 4B), immune score (*r* = 0.386; Figure 4C), and stromal score (*r* = 0.438; Figure 4D). Furthermore, higher risk scores were also associated with increased infiltration of several cell types, most prominently macrophages (*r* = 0.524; Figure 4E), as well as fibroblasts and endothelial cells, suggesting a link between risk stratification and the immune-stromal landscape of glioma.

Furthermore, our analysis revealed positive associations between the risk score and the expression levels of various immune checkpoints across the three datasets (Figure 4F). Notably, these included *SIGLEC7* (*r* = 0.478 in TCGA-LGGGBM, Figure 4G), *PDCD1* (*r* = 0.425 in TCGA-LGGGBM, Figure 4H), *LILRB2* (*r* = 0.458 in TCGA-LGGGBM, Supplementary Figure S6A), *HAVCR2* (*r* = 0.401 in TCGA-LGGGBM, Supplementary Figure S6B), *LAG3* (*r* = 0.425 in TCGA-LGGGBM, Supplementary Figure S6C), and *SIRPA* (*r* = −0.242 in TCGA-LGGGBM, Supplementary Figure S6D). These findings shed light on the intricate interplay between ERGs and the immune microenvironment in glioma.

### 3.5 The correlation of ERGs risk score with both anti-tumor drug sensitivity and the expression of oncogenes

**Figure 5:**
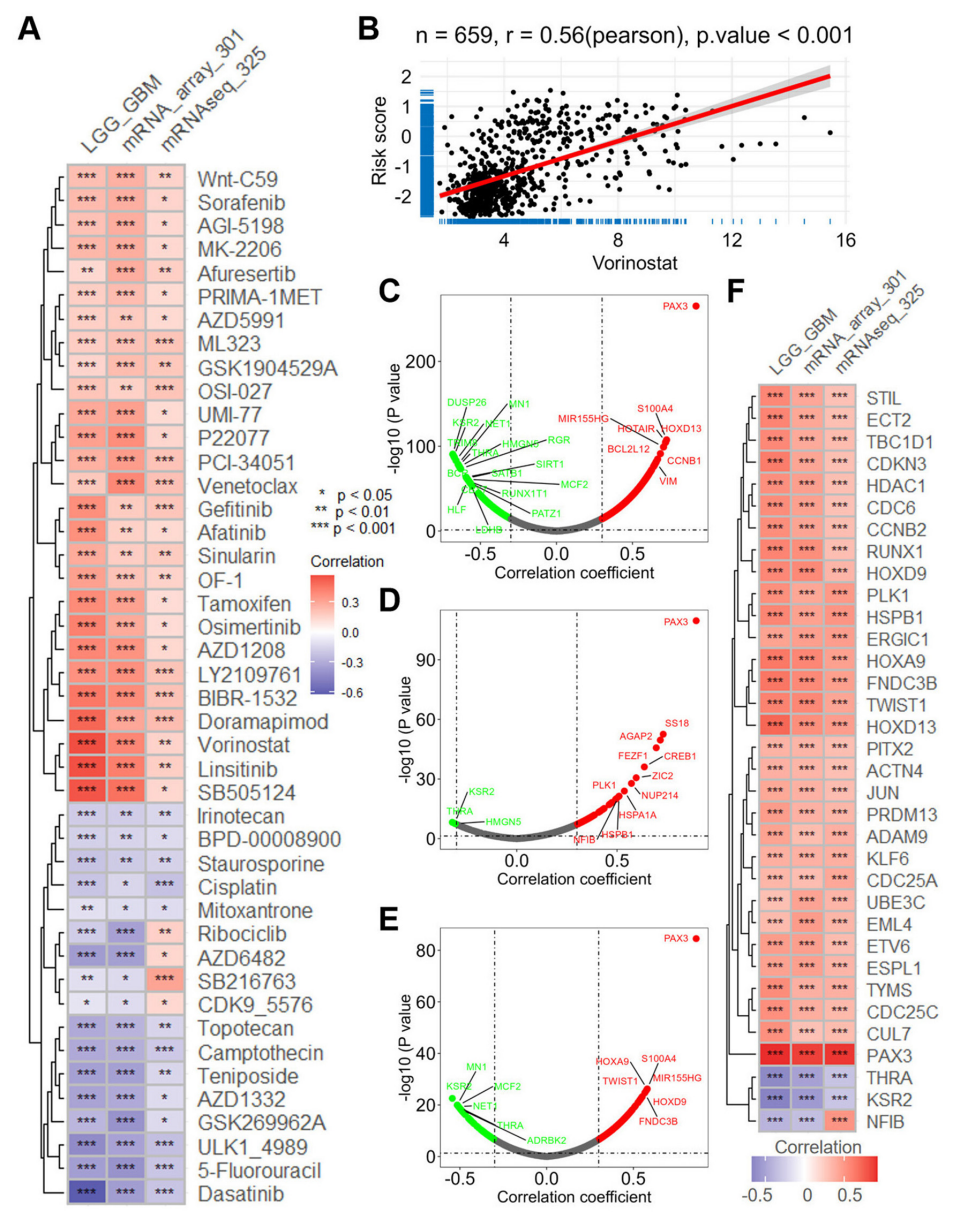
The intricate connections between ERG risk score and the imputed sensitivity of anti-tumor drugs and oncogene expressions. **(A)** Heatmap visualizing the correlation between the risk score and the imputed sensitivity of anti-tumor drugs across the TCGA-LGGGBM dataset and two CGGA datasets. **(B)** Scatter plots offering a focused view of the correlation between the risk score and the sensitivity of vorinostat, a representative anti-tumor drug. **(C–E)** Volcano plots depicting correlation analyses between the risk score and the expression of oncogenes across the TCGA-LGGGBM, mRNAseq_325, and mRNA-array_301 datasets, highlighting significant associations. **(F)** Heatmap presenting the correlation between the risk score and the expression of oncogenes across the TCGA-LGGGBM dataset and two CGGA datasets. CGGA, Chinese Glioma Genome Atlas; ERG, epilepsy-related gene; GBM, glioblastoma; LGG, lower grade glioma; TCGA-LGGGBM, The Cancer Genome Atlas Merged Cohort of LGG and GBM.

Utilizing the “Oncopredict” package and the GDSC V2 database, we predicted the sensitivity score of anti-tumor drugs across three databases, revealing a consistent correlation pattern (Figure 5A). Among the 198 drugs analyzed, several exhibited a positive correlation with the risk score, including vorinostat (*r* = 0.560 in TCGA-LGGGBM, Figure 5B), linsitinib (*r* = 0.553 in TCGA-LGGGBM, Supplementary Figure S7A), doramapimod (*r* = 0.495 in TCGA-LGGGBM, Supplementary Figure S7B), osimertinib (*r* = 0.414 in TCGA-LGGGBM, Supplementary Figure S7C), afatinib (*r* = 0.367 in TCGA-LGGGBM, Supplementary Figure S7D), tamoxifen (*r* = 0.385 in TCGA-LGGGBM, Supplementary Figure S7E), and gefitinib (*r* = 0.370 in TCGA-LGGGBM, Supplementary Figure S7F). Conversely, several drugs demonstrated a negative correlation with the risk score, including dasatinib (*r* = −0.630 in TCGA-LGGGBM, Supplementary Figure S7G), 5-fluorouracil (*r* = −0.358 in TCGA-LGGGBM, Supplementary Figure S7H), AZD1332 (*r* = −0.339 in TCGA-LGGGBM, Supplementary Figure S7I), and teniposide (*r* = −0.350 in TCGA-LGGGBM, Supplementary Figure S7J).

In addition, correlation analyses across all three datasets revealed a consistent positive relationship between the risk score and the expression levels of several well-characterized oncogenes (Figures 5C–F). Specifically, strong associations were observed in the TCGA-LGGGBM cohort for PAX3 (*r* = 0.918, Supplementary Figure S8A), HOXD13 (*r* = 0.717, Supplementary Figure S8B), HOXA9 (*r* = 0.644, Supplementary Figure S8C), and TWIST1 (*r* = 0.598, Supplementary Figure S8D). Similar positive correlations were also noted for RUNX1 (*r* = 0.591, Supplementary Figure S8E), HSPB1 (*r* = 0.586, Supplementary Figure S8F), HDAC1 (*r* = 0.537, Supplementary Figure S8G), and JUN (*r* = 0.421, Supplementary Figure S8H). These findings underscore the intricate associations between the ERG risk score, drug sensitivity, and oncogene expression in glioma.

### 3.6 Correlation of risk score of ERGs with tumor progression

**Figure 6:**
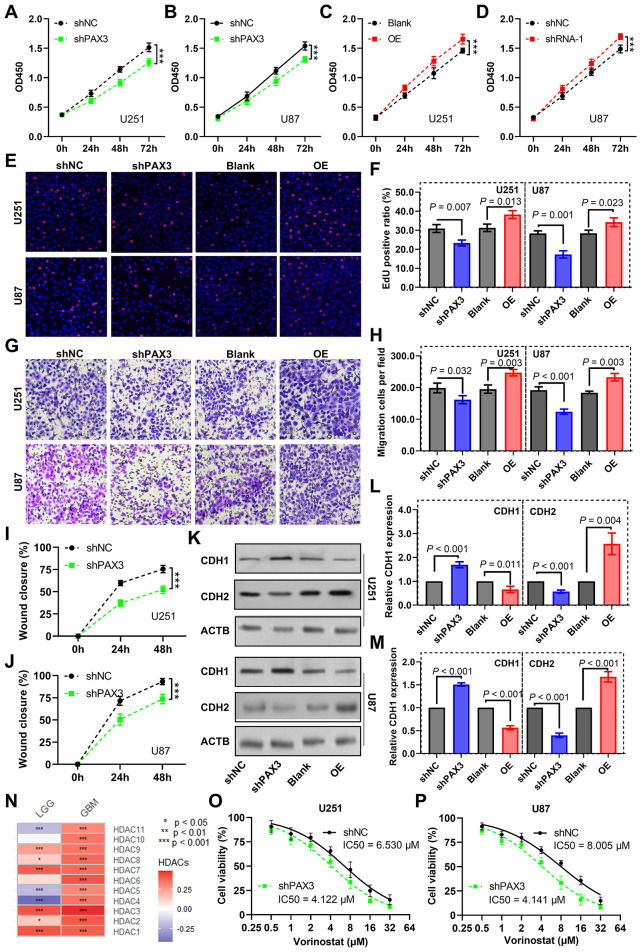
Correlation of the ERG risk score with cancer progression. **(A)** Heatmap illustrating the outcomes of GSEA analysis concerning the biological processes associated with the risk score, utilizing data from TCGA-LGGGBM and two CGGA datasets. **(B)** GSEA plots demonstrating the relationship between risk score and several immune functions such as mononuclear cell differentiation, leukocyte-mediated immunity, and lymphocyte-mediated immunity. **(C)** Heatmap presenting the results of GSEA analysis for KEGG pathways associated with the risk score, using data from TCGA-LGGGBM and two CGGA datasets. **(D)** GSEA plots depicting the association between the risk score and DNA replication, cell cycle, and focal adhesion. Lollipop plots represent the findings of enrichment analysis regarding differentially expressed genes in both biological processes **(E)** and KEGG pathways **(F)** between high- and low-risk groups. CGGA, Chinese Glioma Genome Atlas; ERGs, epilepsy-related genes; GBM, glioblastoma; GSEA, gene set enrichment analysis; KEGG, Kyoto Encyclopedia of Genes and Genomes; LGG, lower grade glioma; TCGA-LGGGBM, The Cancer Genome Atlas Merged Cohort of LGG and GBM.

The risk score of ERGs was found to be closely associated with cancer progression, as indicated by the results of GSEA analysis (Figure 6A). Notably, this risk score exhibited significant correlations with key cancer-related biological processes, including lymphocyte-mediated immunity [normalized enrichment score (NES) = 2.610] and leukocyte-mediated immunity (NES = 2.492). Additionally, it was also linked to mononuclear cell differentiation (NES = 2.157), positive regulation of cell adhesion (NES = 2.216), and mitotic nuclear division (NES = 1.908). All of the above-mentioned processes are presented in Figures 6B–D and Supplementary Figure S9A. Furthermore, the analysis revealed associations with crucial KEGG pathways such as cell cycle (NES = 2.117) and DNA replication. In addition, pathways like the P53 signaling pathway (NES = 1.877) and JAK STAT signaling pathway (NES = 1.938) were also implicated. All of the above-mentioned processes are presented in Figures 6B–D and Supplementary Figure S9A. Further analysis involved differential expression analysis on high-risk and low-risk samples across three datasets, leading to the identification of DEGs with consistent changes (Supplementary Figure S10D). These DEGs were found to be significantly enriched in processes like extracellular matrix organization, angiogenesis, response to drug, cell migration, and cell adhesin (Figure 6E), as well as pathways including ECM-receptor intersection, GnRH secretion, GABAergic synapse, focal adhesion, and TNF (Figure 6F).

### 3.7 Overexpression of PAX3 in glioma and its association with cancer progression

**Figure 7:**
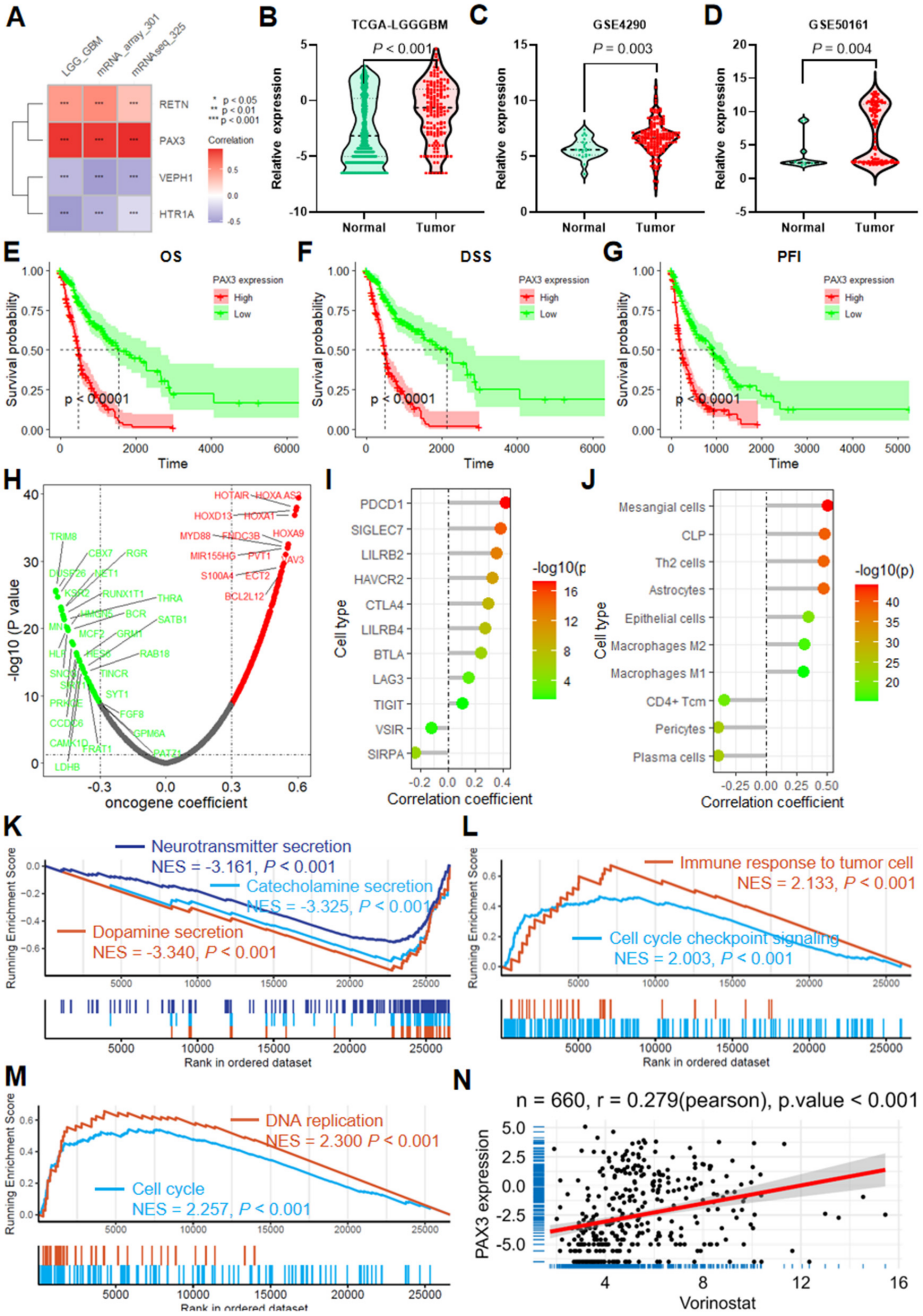
PAX3 expression in glioma and its implications in cancer progression. **(A)** Heatmap illustrating the correlation between ERG risk score and the expression of ERGs within the model. Violin plots displaying the differential expression of *PAX3* in TCGA-LGGGBM **(B)**, GSE4290 **(C)**, and GSE50161 **(D)** datasets, as well as OS analysis results regarding *PAX3* **(E)**, DSS **(F)**, and PFI **(G)** in TCGA-LGGGBM datasets. **(H)** Volcano plot showcasing the correlation between *PAX3* expression and oncogenes. **(I)** Lollipop plot presenting the correlation between *PAX3* expression and immune checkpoint genes. **(J)** Lollipop plot demonstrating the correlation between *PAX3* expression and the infiltration of multiple immune and stroma cells. GSEA plots highlighting the association of *PAX3* with various biological processes **(K, L)** and KEGG pathways **(M)** in the TCGA-LGGGBM dataset. **(N)** Scatter plot depicting *PAX3* expression correlated with the imputed sensitivity score of vorinostat. DSS, disease-specific survival; ERGs, epilepsy-related genes; GBM, glioblastoma; GSEA, gene set enrichment analysis; LGG, lower grade glioma; GBM, Glioblastoma; LGG, lower grade glioma; KEGG, Kyoto Encyclopedia of Genes and Genomes; PFI, progression-free interval; TCGA-LGGGBM, The Cancer Genome Atlas Merged Cohort of LGG and GBM.

In the realm of glioma research, *PAX3* stands out among the ERGs identified in our risk model, boasting the strongest correlation with the risk score (Figure 7A). Analysis across multiple datasets, including TCGA-LGGGBM, GSE4290, and GSE50161, has consistently revealed heightened expression of *PAX3* in glioma tissues compared to their normal counterparts (Figures 7B–D).

Survival analysis within the TCGA-LGGGBM cohort highlighted the clinical relevance of PAX3 expression, revealing that reduced PAX3 levels were significantly associated with progression-free survival, disease-specific survival, and improved overall survival (Figures 7E–G).

Further exploration into its molecular associations unveils intriguing links between *PAX3* expression and various oncogenes, suggesting its potential role as an oncogenic driver in glioma progression (Figure 7H). Moreover, *PAX3* expression demonstrates significant positive correlations with multiple immune checkpoints, particularly *PDCD1, SIGLEC7*, and *LILRB2*, underscoring its involvement in immune regulation within the glioma microenvironment (Figure 7I).

The influence of PAX3 extends beyond molecular signaling, encompassing alterations in the tumor microenvironment, as reflected by its positive correlations with the infiltration of various immune and stromal cell populations, such as mesangial cells, common lymphoid progenitors, Th2 cells, and astrocytes (Figure 7J).

GSEA enrichment analysis provides further insights into the functional relevance of *PAX3* in glioma biology, linking it to key cancer-related biological processes and KEGG pathways such as neurotransmitter secretion, immune response to tumor cells, and cell cycle regulation (Figures 7K–M).

Moreover, *PAX3* expression has emerged as a potential determinant of therapeutic sensitivity, with its levels significantly correlating with the responsiveness to multiple anti-tumor drugs, including vorinostat, doramapimod, linsitinib, and tamoxifen, among others (Figure 7N, Supplementary Figure S11). Conversely, it also demonstrates associations with resistance to certain agents like 5-fluorouracil, dasatinib, and entospletinib, underscoring its multifaceted role in modulating therapeutic responses in glioma (Supplementary Figure S11).

### 3.8 Implication of PAX3 in the cell proliferation, migration, and vorinostat sensitivity of glioma

**Figure 8:**
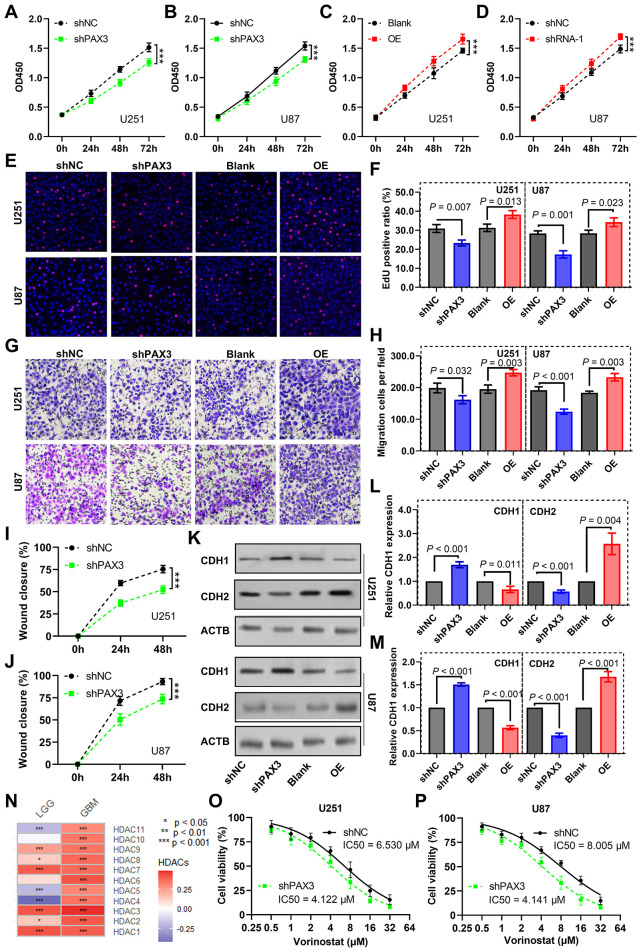
Impact of PAX3 on glioma cell dynamics and vorinostat sensitivity. **(A–D)** CCK-8 assay evaluating the effects of *PAX3* knockdown or overexpression on U251 and U87 cell proliferation. **(E)** Images representing cell proliferation in the EdU assay and **(F)** corresponding quantitative analysis exhibiting changes in cell proliferation of glioma cells upon knockdown or overexpression of *PAX3*. **(G)** Images representing migration status in transwell cell migration assay and **(H)** corresponding quantitative analysis exhibiting changes in migration ability of glioma cells upon knockdown or overexpression of *PAX3*. **(I, J)** Quantitative findings delineating alterations in glioma cell migratory capacity following *PAX3* knockdown in the wound healing assay. **(K)** Images representing expression of E-cadherin and *CDH2* in glioma cells by western blot analysis and **(L, M)** corresponding quantitative analysis results demonstrating alterations in their expression in glioma cells following *PAX3* knockdown or overexpression. **(N)** Heatmap depicting the association between *PAX3* expression levels and *HDACs*. **(O, P)** CCK-8 assay measuring the IC50 of vorinostat in glioma cells with *PAX3* knockdown or overexpression. CCK-8, Cell Counting Kit-8; *CDH1*, E-cadherin; *CDH2*, N-cadherin; IC50, half-maximal inhibitory concentration.

To investigate *PAX3*'s role in glioma cell dynamics, we manipulated its expression using shRNA and overexpression plasmids (Supplementary Figure S12). The results from the CCK-8 assay unveiled a significant inhibition of cell proliferation of U251 (Figure 8A) and U87 cells (Figure 8B) upon *PAX3* knockdown, while overexpression of *PAX3* notably promoted glioma cell proliferation (Figures 8C, D). Consistent findings were observed in the EdU assay, where *PAX3* knockdown attenuated glioma cell proliferation, whereas its overexpression augmented this process (Figures 8E, F).

Moreover, we performed a transwell migration assay. The results demonstrated that *PAX3* knockdown markedly reduced glioma cell migration, whereas overexpression enhanced it (Figures 8G, H). Similarly, wound scratch healing assays revealed a decelerated wound closure rate in glioma cells upon *PAX3* knockdown (Figures 8I, J).

At the molecular level, western blot analysis showed that *PAX3* knockdown led to a downregulation of E-cadherin expression (an epithelial marker) and an upregulation of CDH2 (a mesenchymal marker). Conversely, *PAX3* overexpression induced opposite changes (Figures 8K–M).

Furthermore, we explored the impact of *PAX3* on vorinostat (a histone deacetylase inhibitor) sensitivity. Correlation analysis indicated a positive association between *PAX3* expression and several vorinostat targets, particularly *HDAC1*/*2/3* (Figure 8N). Intriguingly, CCK-8 assays revealed that *PAX3* knockdown increased the sensitivity of U251 (Figure 8O) and U87 (Figure 8P) cells to vorinostat, highlighting a potential role of *PAX3* in modulating drug sensitivity in glioma cells.

## 4 Discussion

Glioma remains one of the most aggressive and treatment-resistant tumors of the central nervous system, with glioblastoma representing the most lethal subtype (18). Despite multimodal treatment strategies, including surgery, radiation, and chemotherapy, prognosis remains poor due to significant inter- and intra-tumoral heterogeneity, frequent recurrence, and treatment resistance (18–20). These challenges highlight the urgent need for more effective tools to stratify patients and to discover novel therapeutic targets to improve clinical outcomes.

One underexplored aspect of glioma is its association with epilepsy, which frequently occurs as an early clinical manifestation and may carry prognostic significance. However, the molecular mechanisms linking epilepsy-related pathways and glioma progression remain largely unclear (21, 22). In this study, we systematically analyzed glioma transcriptomic data stratified by epilepsy status and identified differentially expressed epilepsy-related genes (ERGs). Through rigorous statistical modeling—including univariate Cox, LASSO, and multivariate Cox regression—we developed a four-gene prognostic model consisting of *PAX3, RETN, VEPH1*, and *HTR1A*. This model showed excellent prognostic discrimination in both internal (TCGA) and external (CGGA) validation cohorts, with area under the curve (AUC) values exceeding 0.85 in the training set and remaining above 0.73 in independent datasets.

Importantly, this ERG-based model not only stratifies glioma patients into high- and low-risk groups with distinct overall survival outcomes but also correlates with immune infiltration levels and the expression of multiple immune checkpoint genes, such as *PDCD1, SIGLEC7*, and *LILRB2*. This suggests that the ERG signature may reflect broader tumor-immune interactions, thereby contributing to both prognosis and treatment responsiveness. The inclusion of epilepsy-related molecular features represents a novel direction for prognostic modeling in glioma and may provide new biological insights into seizure-associated gliomagenesis.

Beyond prognostic value, our study also sheds light on potential therapeutic vulnerabilities, particularly focusing on *PAX3*. Among the four ERGs, *PAX3* showed the strongest correlation with the risk score and was consistently overexpressed in glioma tissues across datasets. Functional experiments demonstrated that *PAX3* promotes glioma cell proliferation and migration, accompanied by modulation of epithelial–mesenchymal transition (EMT) markers (23, 24).

An important focus in tumor microenvironment research is how changes in immune cell composition and immune checkpoint activity affect tumor progression. In glioma, certain immune cells, such as neutrophils, macrophages, activated dendritic cells, and activated CD4+ T cells, are linked to worse outcomes (25). In line with this, our study found that higher ERG-based risk scores were associated with increased infiltration of macrophages, tumor-associated fibroblasts, and endothelial cells (26, 27). We also observed positive correlations between the risk score and several immune checkpoint genes, including *SIGLEC7, PDCD1, LILRB2, HAVCR2*, and *LAG3*, which have been tied to poor prognosis in glioma (27). These results support the idea that targeting the immune microenvironment and immune checkpoints may help improve glioma treatment outcomes (28).

Postoperative chemoradiotherapy remains the standard treatment for glioma, especially glioblastoma. Temozolomide (TMZ), an oral alkylating agent, plays a key role by inducing tumor cell death through DNA methylation. Its good tolerability, ability to cross the blood-brain barrier, and high oral bioavailability make it the first-line drug for glioma (29). However, its effectiveness is often short-lived, as glioma cells commonly develop resistance within a few months. This highlights the urgent need to investigate resistance mechanisms and identify new therapeutic targets to improve treatment outcomes.

PAX3 is a crucial stem cell transcription factor involved in embryonic development and plays a role in neural cell differentiation by downregulating glial fibrillary acidic protein. Previous research has shown that silencing *PAX3* can suppress tumor growth in several cancers, including gastric and prostate cancer, supporting its role as a potential oncogene (30, 31). In our study, elevated *PAX3* expression in glioma was associated with enhanced cell proliferation, migration, and reduced sensitivity to the histone deacetylase inhibitor vorinostat. Mechanistically, *PAX3* expression positively correlated with HDAC1/2/3 levels, and its knockdown increased glioma cell sensitivity to vorinostat. These results indicate that *PAX3* not only promotes glioma progression but also contributes to therapeutic resistance, positioning it as a promising target for glioma therapy.

## 5 Limitations

This study has several limitations. The prognostic model was developed using datasets that included both IDH-mutant and IDH–wild-type gliomas; although it showed strong performance overall, further validation in IDH–wild-type glioblastomas, which represent a distinct and aggressive subgroup, is needed. PAX3 emerged as a key regulator of glioma migration and vorinostat response, yet its function may vary across molecular subtypes such as mesenchymal and proneural. While our assays support a role for PAX3 in proliferation, migration, and drug sensitivity, the underlying mechanisms remain unclear and require further dissection of its interactions with subtype-specific pathways. In addition, advanced tools such as single-cell RNA sequencing and machine learning, which have proven valuable for capturing glioma heterogeneity and progression (2, 3), were not extensively applied here; incorporating them could refine both mechanistic insight and model accuracy. Finally, our functional work was limited to two established glioma cell lines (U87 and U251), which do not reflect the full diversity of gliomas. Broader validation in additional models, including patient-derived glioma cells and xenografts, will be essential. Despite these limitations, our study presents a validated four-gene ERG-based model and highlights PAX3 as a potential therapeutic target, providing a foundation for more personalized glioma therapy.

## Data Availability

The raw data supporting the conclusions of this article will be made available by the authors, without undue reservation.
