# Supplementary material for: Prognostic modeling of glioma using epilepsy-related genes highlights PAX3 as a regulator of migration and vorinostat sensitivity
Source: Front Neurol. 2025 Oct 17;16:1665835. doi: 10.3389/fneur.2025.1665835 (PMC12576345; doi:10.3389/fneur.2025.1665835)
Supplement: Supplementary file 1 [file Supplementary_file_1.docx]

Supplementary Tables

Table S1, Description of data sources, quality control and sequencing platform differences

|  | Source | Cohort Role | Platform/Type | Data Format (Normalized) |
| --- | --- | --- | --- | --- |
| TCGA_LGG | UCSC Xena（TCGA HiSeqV2） | Epilepsy-related gene discovery | Illumina HiSeq RNA-seq | RSEM normalized，log2(x+1) |
| TCGA_LGGGBM | UCSC Xena（TCGA HiSeqV2） | Model training | Illumina HiSeq RNA-seq | RSEM normalized，log2(x+1) |
| GSE199759 | GEO | Epilepsy-related gene discovery | Illumina RNA-seq | Processed matrix provided by the platform (log transformation as needed) |
| mRNAseq_325 | CGGA | External validation | Illumina HiSeq RNA-seq | FPKM，log2(FPKM+1) |
| mRNA-array_301 | CGGA | External validation | Agilent Whole Human Genome Microarray | Quantile normalization, log2; multiple probes summarized at the gene level |

**Table S2.** Distribution of clinicopathological features in the training and internal testing datasets.

| Characteristics | | Training | Internal test | All | χ2 test p |
| --- | --- | --- | --- | --- | --- |
| Age |  | 46.53 ± 14.96 | 47.09 ± 15.61 | 46.75 ± 15.21 | 0.642 |
| Gender | FEMALE | 161 (40.66%) | 117 (44.49%) | 278 (42.19%) | 0.622 |
|  | MALE | 235 (59.34%) | 146 (55.51%) | 381 (57.81%) | |
| IDH_mutation_found | NO | 16 (20.51%) | 18 (40.00%) | 34 (27.64%) | 0.067 |
|  | YES | 62 (79.49%) | 27 (60.00%) | 89 (72.36%) | |
| Grade | G2 | 148 (47.90%) | 98 (49.75%) | 246 (48.62%) | 0.921 |
|  | G3 | 161 (52.10%) | 99 (50.25%) | 260 (51.38%) | |
| time |  | 2.43 ± 2.55 | 2.09 ± 2.28 | 2.29 ± 2.45 | 0.081 |
| status | 0 | 249 (62.88%) | 168 (63.88%) | 417 (63.28%) | 0.967 |
|  | 1 | 147 (37.12%) | 95 (36.12%) | 242 (36.72%) | |

**Table S3.** Distribution of clinicopathological features in the external validation datasets.

| Characteristics | | mRNA-array_301 | mRNAseq_325 | χ2 test p |
| --- | --- | --- | --- | --- |
| Grade | WHO II | 105 (37.37%) | 98 (31.72%) | 0.207 |
|  | WHO III | 53 (18.86%) | 74 (23.95%) | |
| Grade | WHO IV | 123 (43.77%) | 137 (44.34%) | |
| Gender | Female | 117 (41.20%) | 116 (37.06%) | 0.342 |
|  | Male | 167 (58.80%) | 197 (62.94%) | |
| Age |  | 42.68 ± 11.69 | 43.17 ± 11.98 | 0.615 |
| IDH_mutation_status | Mutant | 126 (44.52%) | 167 (53.53%) | 0.035 |
|  | Wildtype | 157 (55.48%) | 145 (46.47%) | |
| MGMTp_methylation_status | methylated | 95 (34.67%) | 152 (51.53%) | <0.001 |
|  | un-methylated | 179 (65.33%) | 143 (48.47%) | |
| time |  | 4.53 ± 4.39 | 3.98 ± 4.03 | 0.110 |
| status | 0 | 99 (34.86%) | 95 (30.35%) | 0.277 |
|  | 1 | 185 (65.14%) | 218 (69.65%) | |

**Table S4.** Distribution of risk scores across different clinicopathological characteristics in the TCGA_LGGGBM dataset.

| Characteristics | | N | Mean ± SD | P value |
| --- | --- | --- | --- | --- |
| Age | > 40 | 394 | -0.837 ± 1.125 | < 0.001 |
|  | ≤ 40 | 265 | -1.632 ± 0.793 | |
| Gender | FEMALE | 278 | -1.206 ± 1.039 | 0.320 |
|  | MALE | 381 | -1.122 ± 1.104 | |
| KPS.score | 40 | 9 | -0.009 ± 0.981 | < 0.001 |
|  | 50 | 8 | -1.330 ± 1.242 | |
|  | 60 | 36 | -0.126 ± 0.928 | |
|  | 70 | 29 | -1.140 ± 0.957 | |
|  | 80 | 118 | -0.530 ± 1.123 | |
|  | 90 | 122 | -1.523 ± 0.865 | |
|  | 100 | 90 | -1.530 ± 0.930 | |
| IDH_mutation_found | NO | 34 | -0.891 ± 0.999 | < 0.001 |
|  | YES | 89 | -1.798 ± 0.717 | |
| Grade | G2 | 246 | -1.801 ± 0.629 | < 0.001 |
|  | G3 | 260 | -1.316 ± 0.950 | |

**Table S5.** Distribution of risk scores across different clinicopathological characteristics in the mRNA_array_301 dataset.

| Characteristics | | N | Mean ± SD | P value |
| --- | --- | --- | --- | --- |
| Grade | WHO II | 105 | -0.142 ± 0.276 | < 0.001 |
|  | WHO III | 53 | 0.067 ± 0.299 | |
|  | WHO IV | 123 | 0.209 ± 0.263 | |
| Gender | Female | 117 | 0.059 ± 0.348 | 0.863 |
|  | Male | 167 | 0.052 ± 0.295 | |
| Age | > 40 | 151 | 0.130 ± 0.316 | < 0.001 |
|  | ≥ 40 | 131 | -0.031 ± 0.300 | |
| IDH_mutation_status | Mutant | 126 | -0.100 ± 0.281 | < 0.001 |
|  | Wildtype | 157 | 0.181 ± 0.289 | |
| MGMTp_methylation_status | methylated | 95 | 0.043 ± 0.375 | 0.645 |
|  | un-methylated | 179 | 0.064 ± 0.292 | |

**Table S6.** Distribution of risk scores across different clinicopathological characteristics in the mRNAseq_325 dataset.

| Characteristics | | N | Mean ± SD | P value |
| --- | --- | --- | --- | --- |
| Grade | WHO II | 98 | -0.452 ± 0.487 | < 0.001 |
|  | WHO III | 74 | 0.037 ± 1.725 | |
|  | WHO IV | 137 | 0.180 ± 0.670 | |
| Gender | Female | 116 | -0.136 ± 0.642 | 0.211 |
|  | Male | 197 | -0.006 ± 1.188 | |
| Age | > 40 | 177 | -0.007 ± 0.593 | 0.399 |
|  | ≥ 40 | 136 | -0.115 ± 1.394 | |
| IDH_mutation_status | Mutant | 167 | -0.381 ± 0.482 | < 0.001 |
|  | Wildtype | 145 | 0.327 ± 1.310 | |
| MGMTp_methylation_status | methylated | 152 | -0.166 ± 0.613 | 0.027 |
|  | un-methylated | 143 | 0.107 ± 1.345 | |

Supplementary Figures


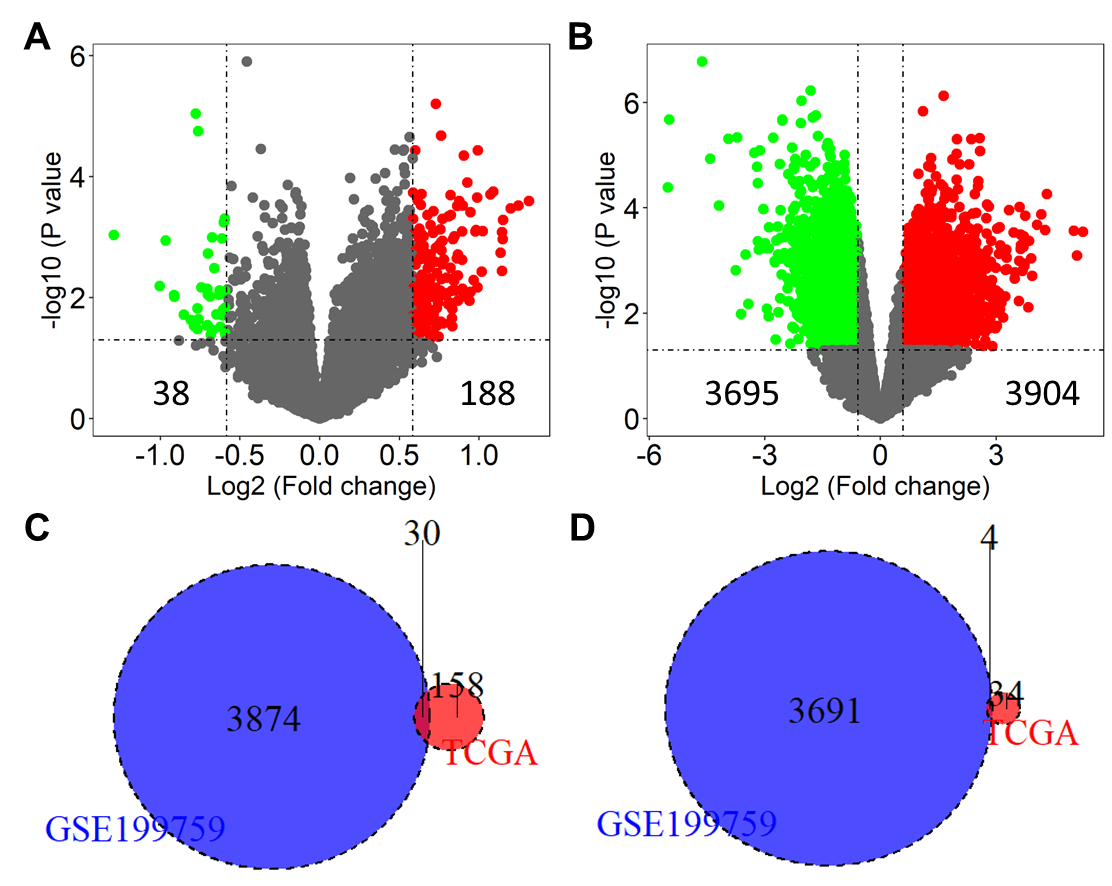


**Figure S1. Screening of epilepsy-related genes (ERGs).** Volcano plots show differentially expressed genes in TCGA_LGG (A) and GSE199759 (B). Venn diagrams show intersecting upregulated (C) and downregulated (D) genes between the two datasets. TCGA: The Cancer Genome Atlas; LGG: low-grade glioma.


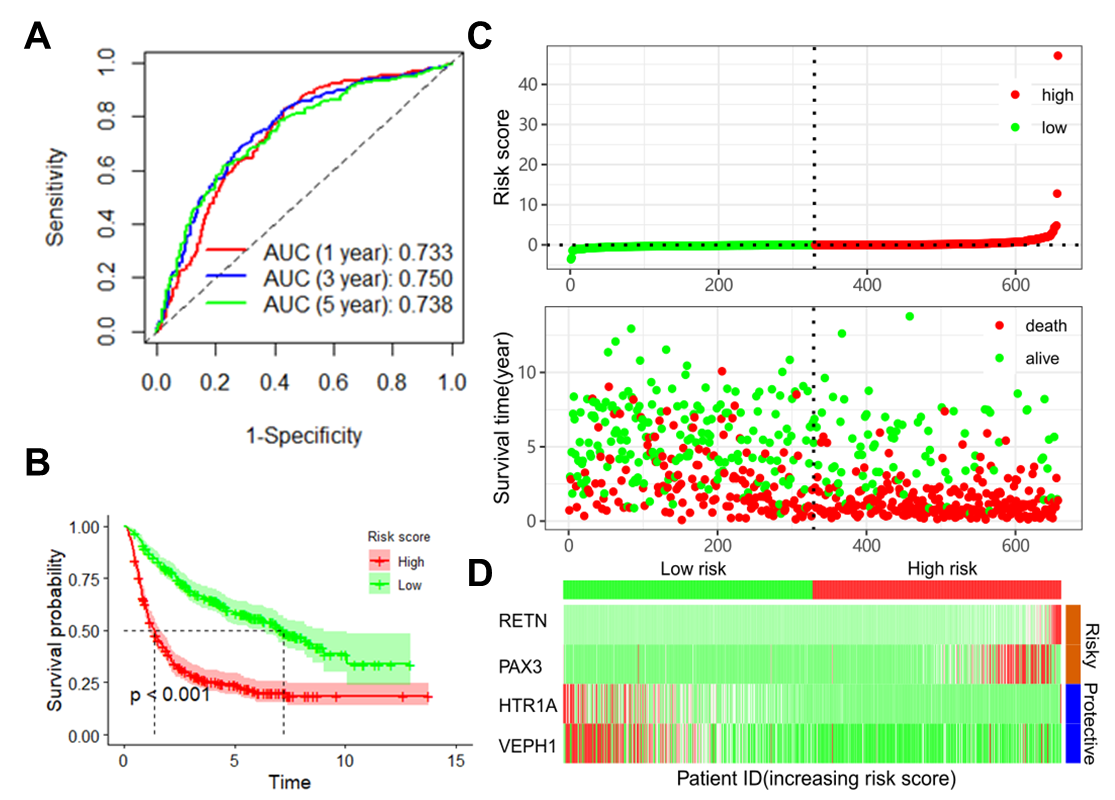


**Figure S2. Validation of the four-ERG prognostic model in the internal testing dataset.** (A) ROC curve for 5-year overall survival. (B) Kaplan–Meier curve for overall survival. (C) Distribution of risk scores and survival status. (D) Heatmap of the expression of four ERGs. ROC: receiver operating characteristic.


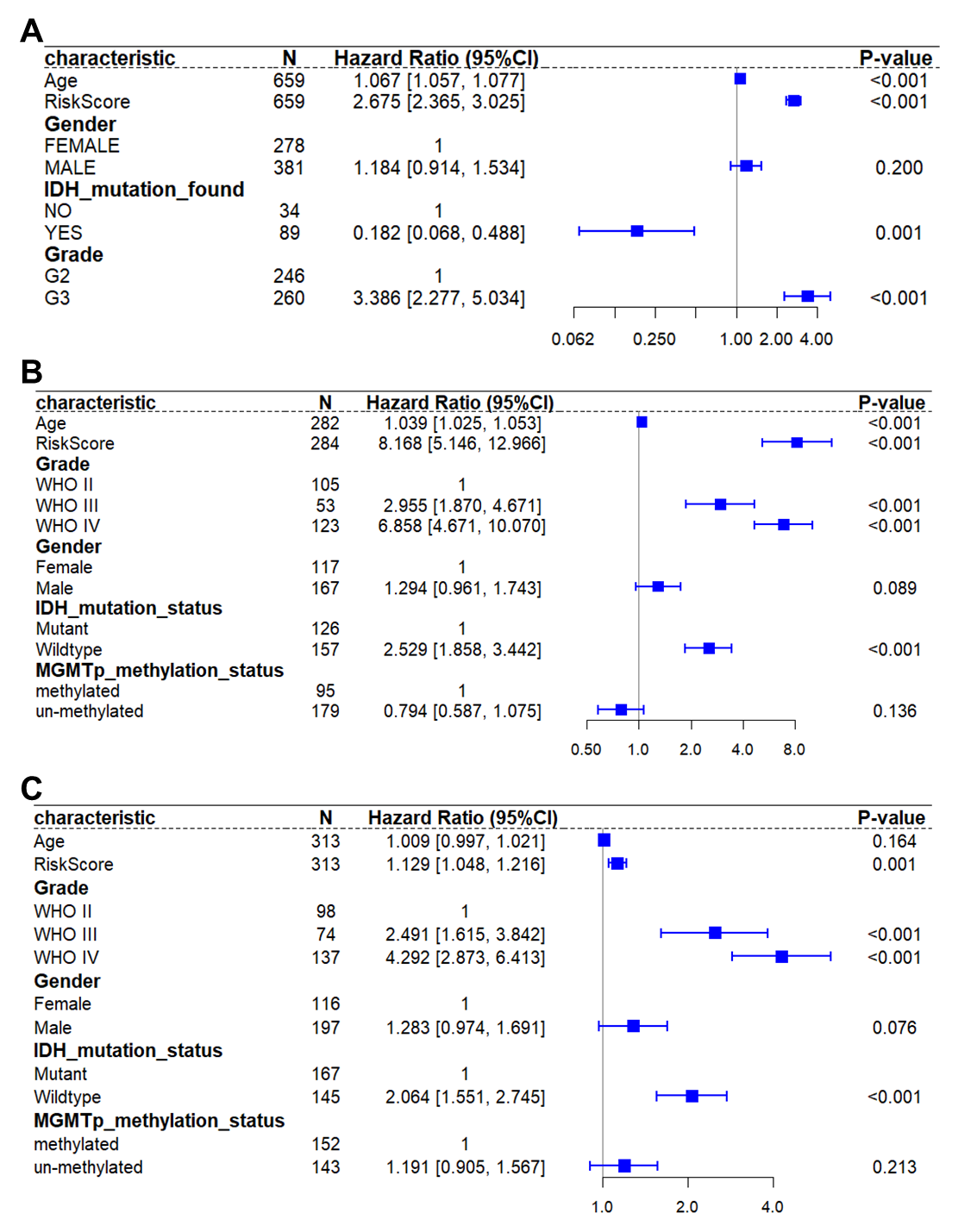


**Figure S3. Univariate Cox analysis of clinical and pathological features across three glioma datasets.** Forest plots show results from TCGA_LGGGBM (A), mRNA_array_301 (B), and mRNAseq_325 (C). TCGA: The Cancer Genome Atlas; LGG: low-grade glioma; GBM: glioblastoma.


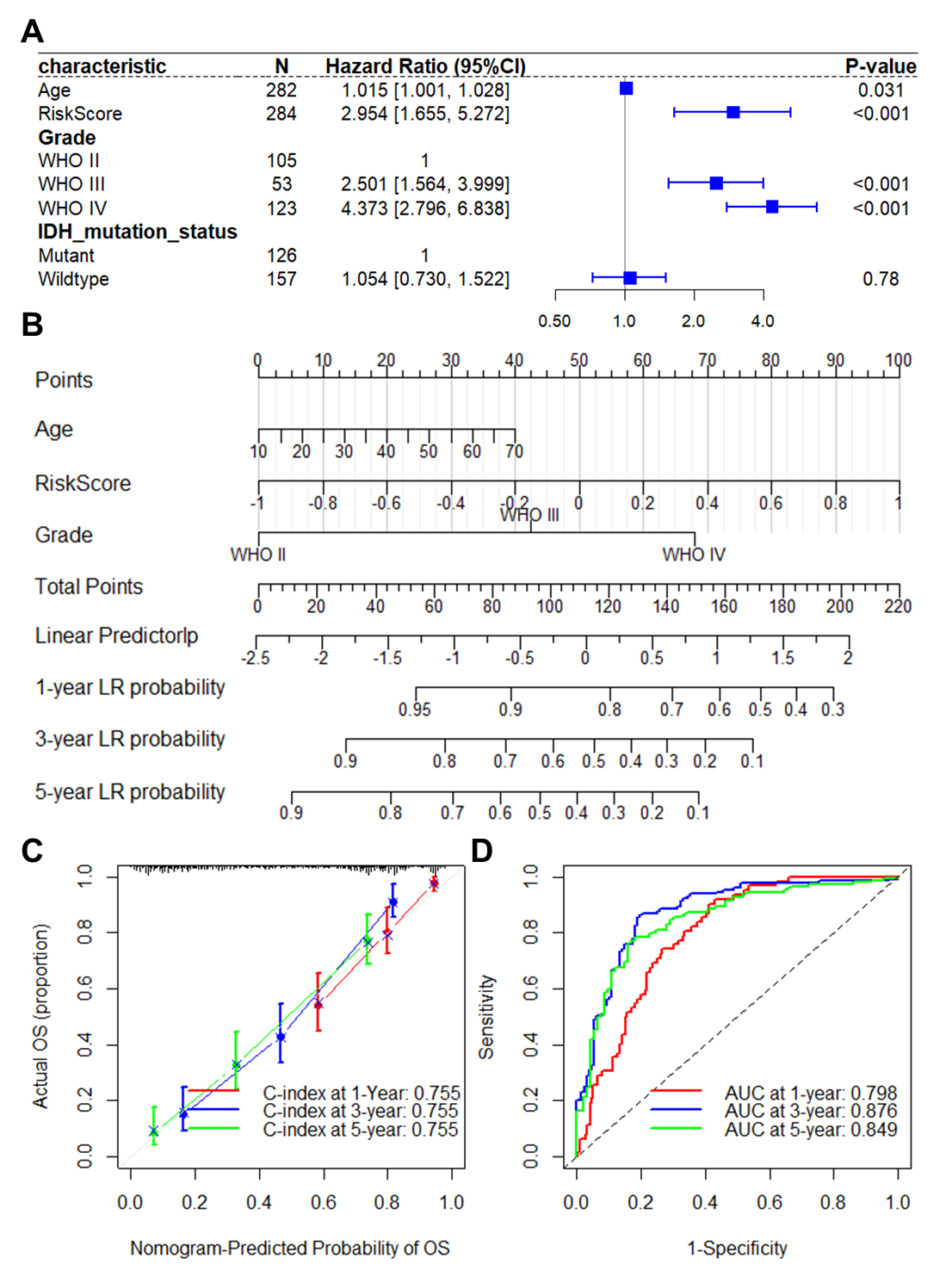


**Figure S4. Prognostic value of ERG-based risk scores in the mRNA_array_301 dataset.** (A) Multivariate Cox regression analysis of risk score and clinical features. (B) Nomogram incorporating risk score and age. (C) Calibration plots for 1-, 3-, and 5-year overall survival. (D) Kaplan–Meier curve based on nomogram-derived scores.


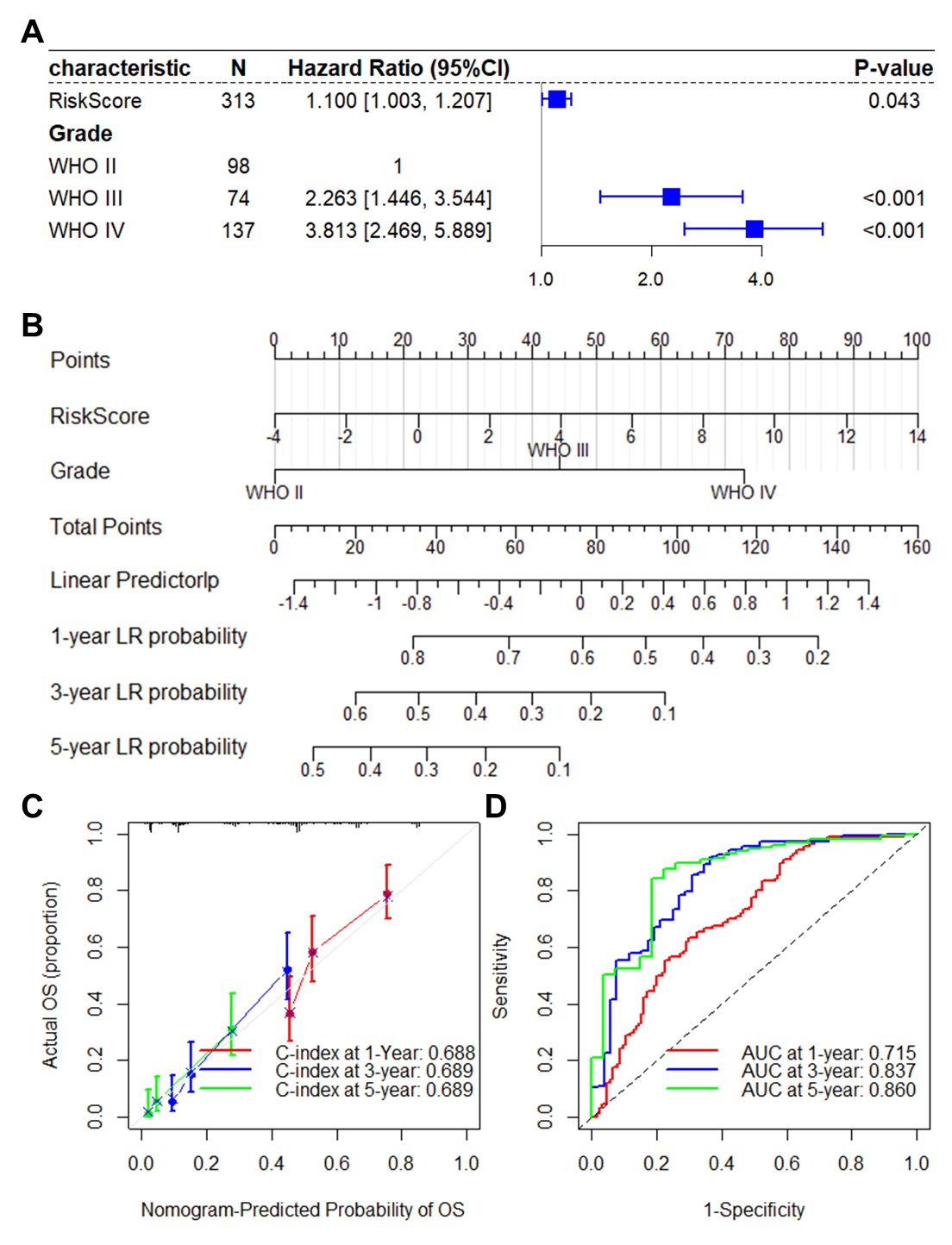


**Figure S5. Prognostic value of ERG-based risk scores in the mRNAseq_325 dataset.** (A) Multivariate Cox regression analysis of risk score and clinical features. (B) Nomogram incorporating risk score and age. (C) Calibration plots for 1-, 3-, and 5-year overall survival. (D) Kaplan–Meier curve based on nomogram-derived scores.


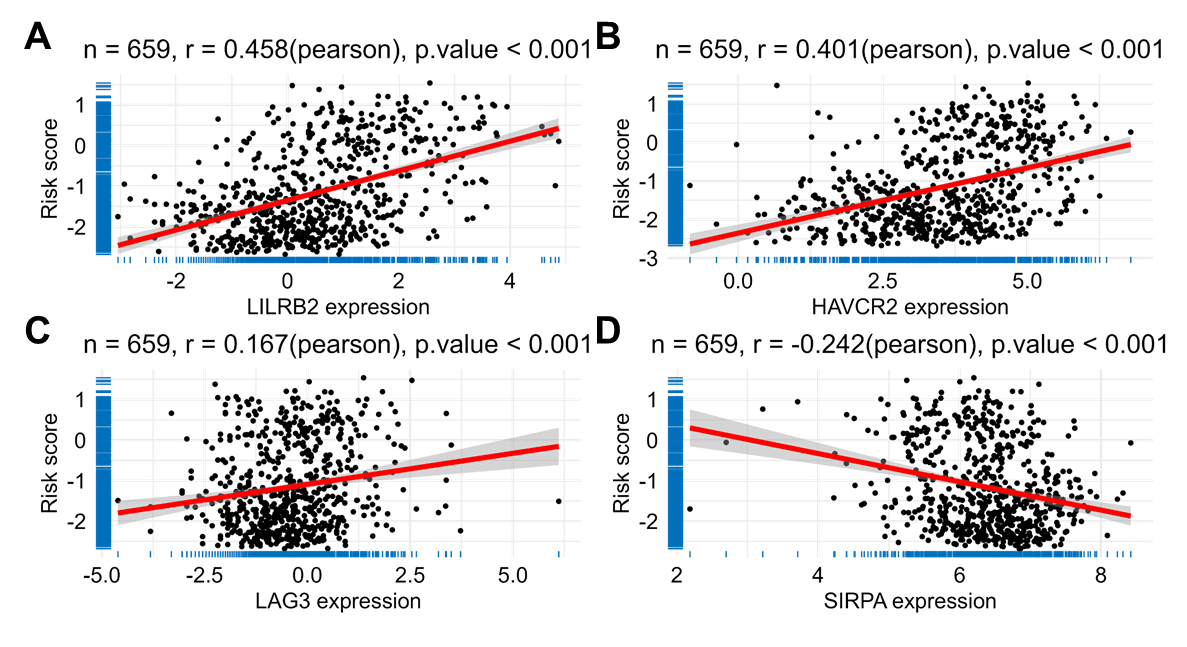


**Figure S6. Correlation between ERG-based risk scores and immune checkpoint gene expression in TCGA_LGGGBM.** Scatter plots show associations with LILRB2 (A), HAVCR2 (B), LAG3 (C), and SIRPA (D). TCGA: The Cancer Genome Atlas. LGG: low-grade glioma. GBM: glioblastoma.


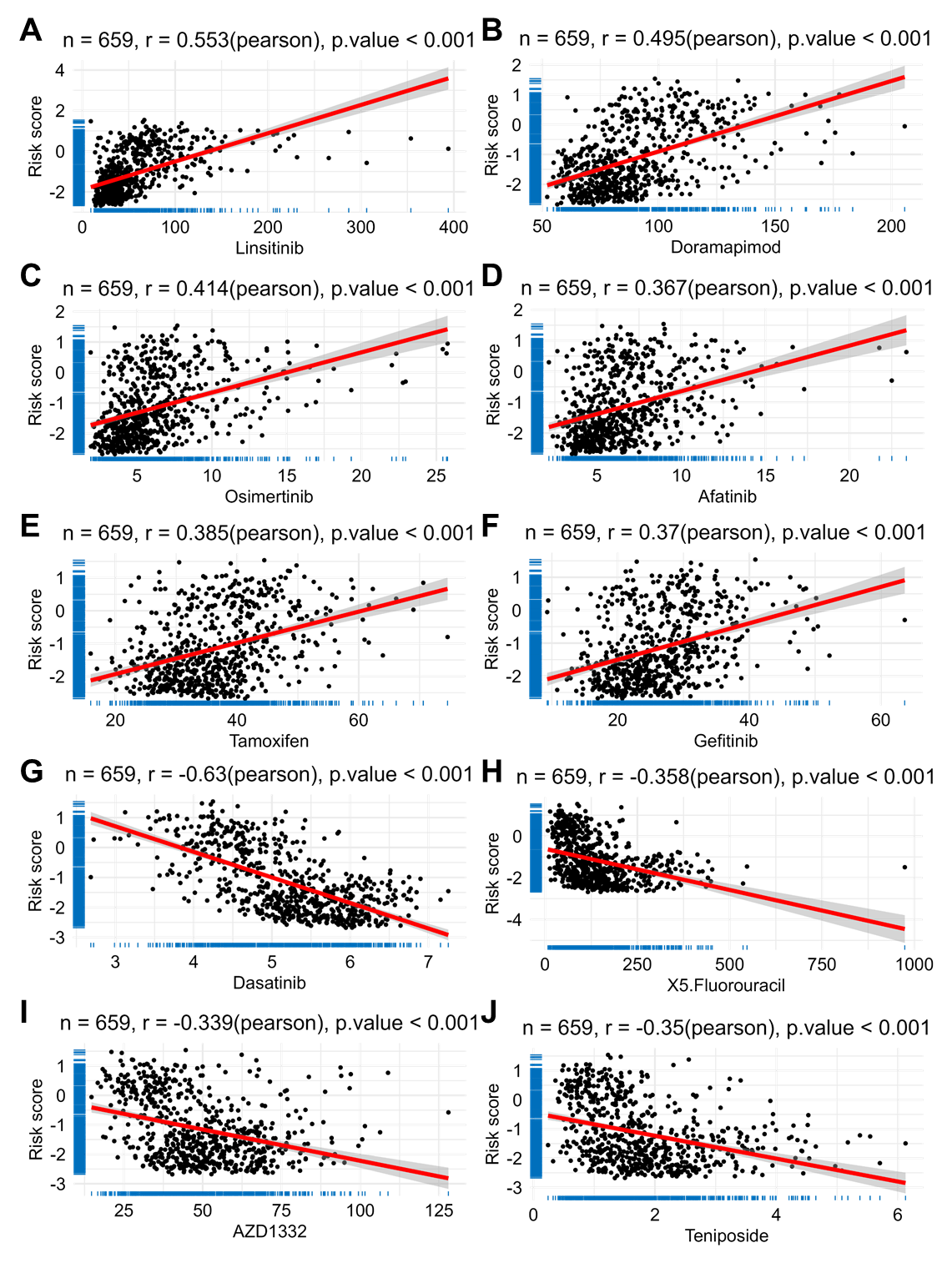


**Figure S7.** **Correlation between ERG-based risk scores and sensitivity to antitumor drugs in TCGA_LGGGBM.**


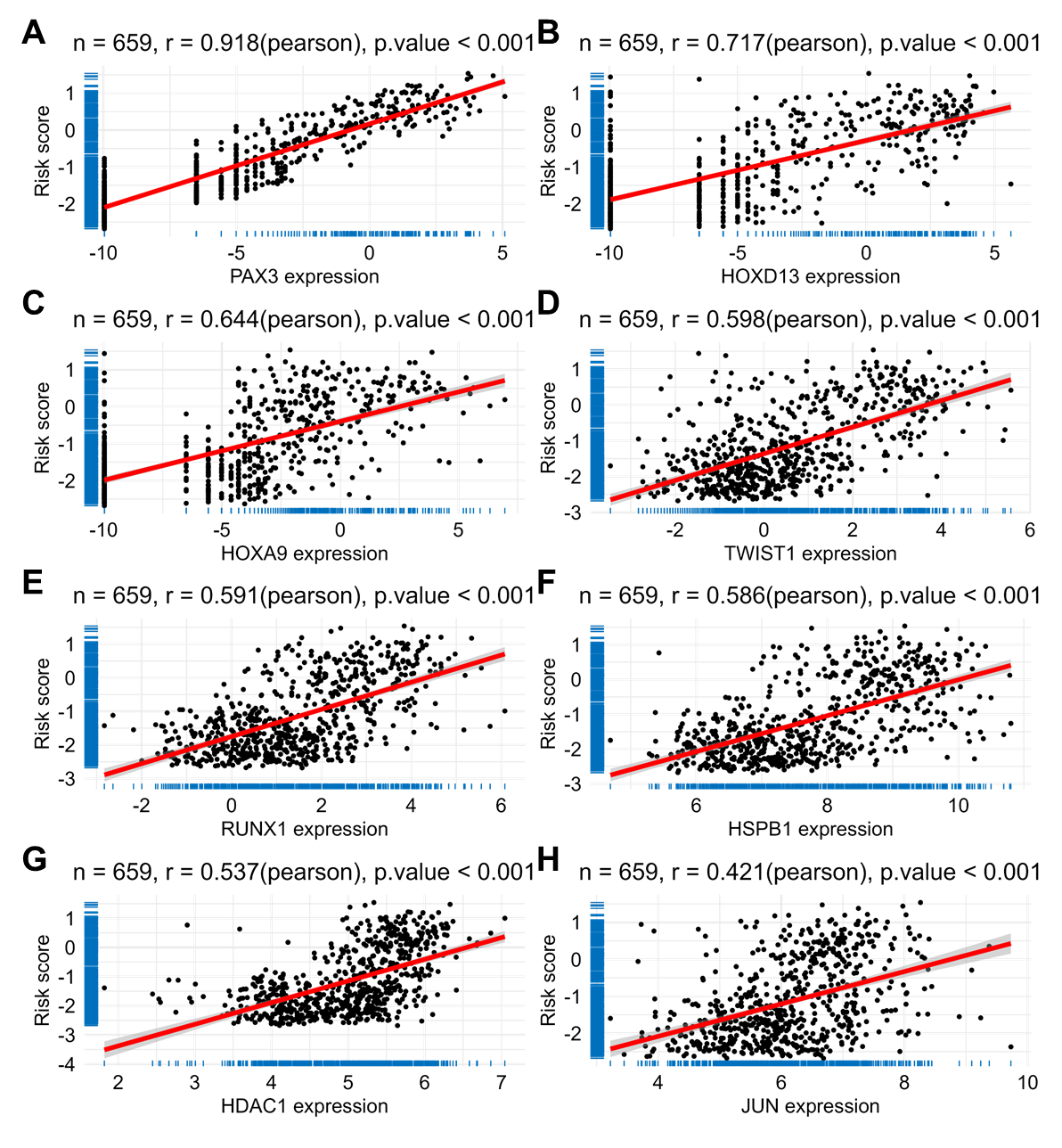


Figure S8. Correlation between ERG-based risk scores and cancer gene expression in TCGA_LGGGBM.


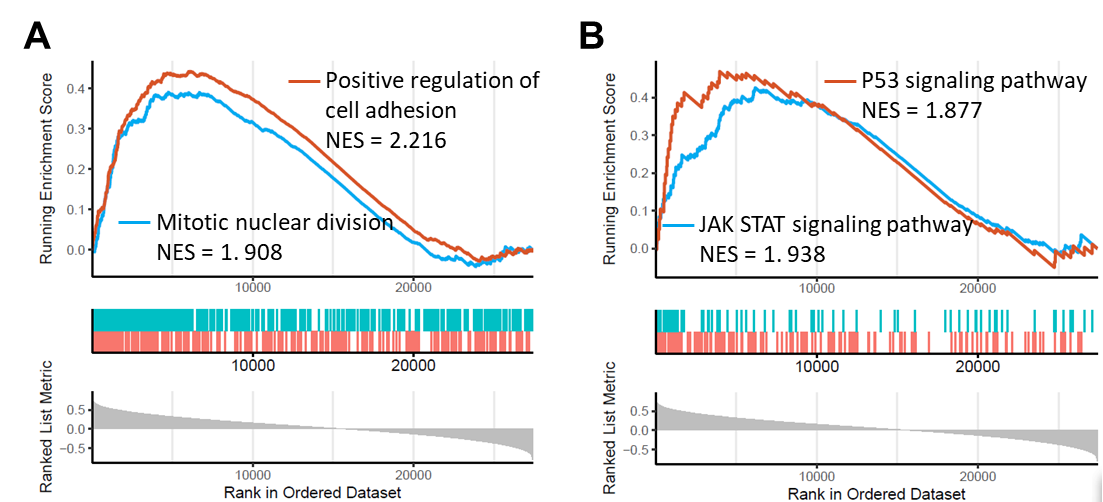


**Figure S9.** **Gene set enrichment analysis (GSEA) of pathways associated with ERG-based risk scores in TCGA_LGGGBM.** Enriched pathways include Positive Regulation of Cell Adhesion and Mitotic Nuclear Division (A), and p53 and JAK-STAT signaling pathways (B).


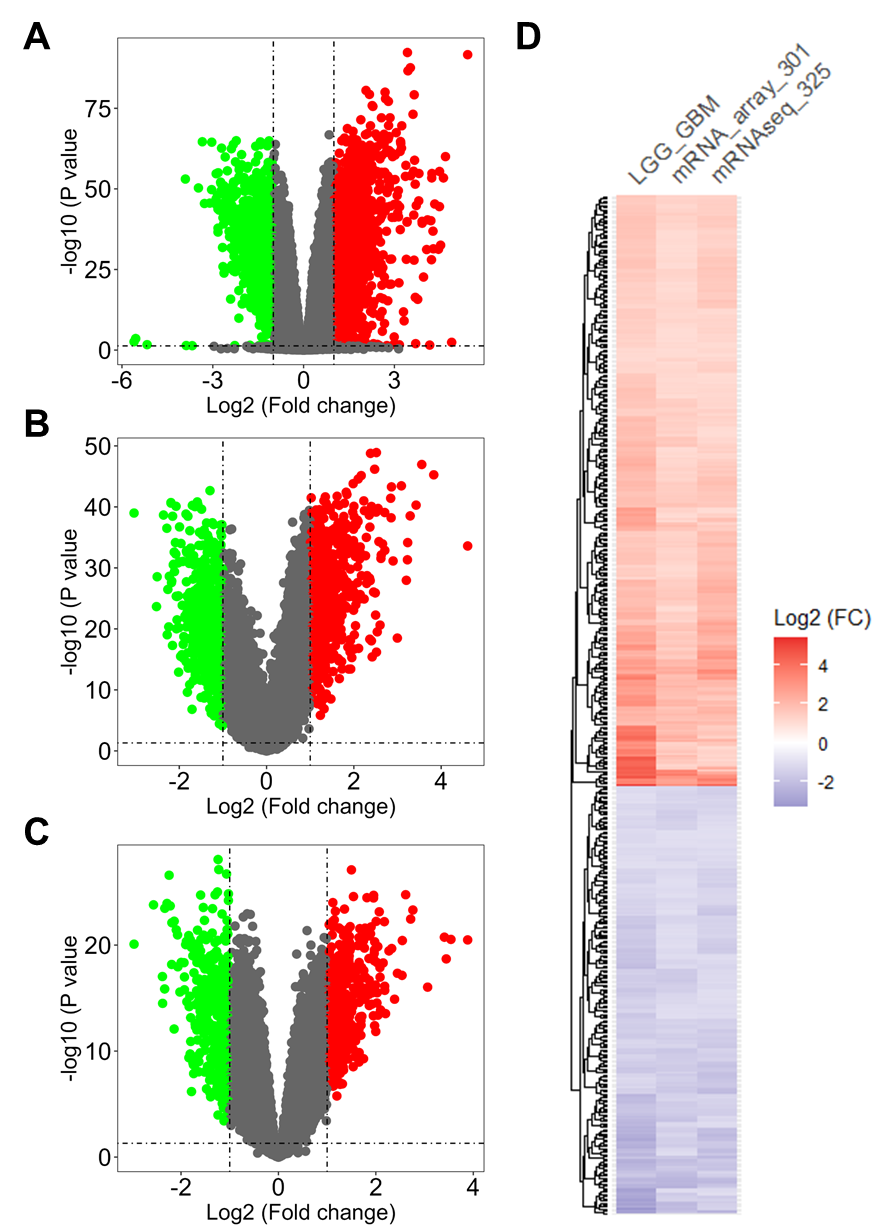


**Figure S10. Different expression genes between high and low risk groups.** The volcanic map shows TCGA-LGGGBM (A) mRNA-RARAY_301, (B) and mRNaseQ_325, (C) Different expression genes of data concentration high and low risk group, and (D) The hot diagram shows the intersection of the three data set differences to express genes.


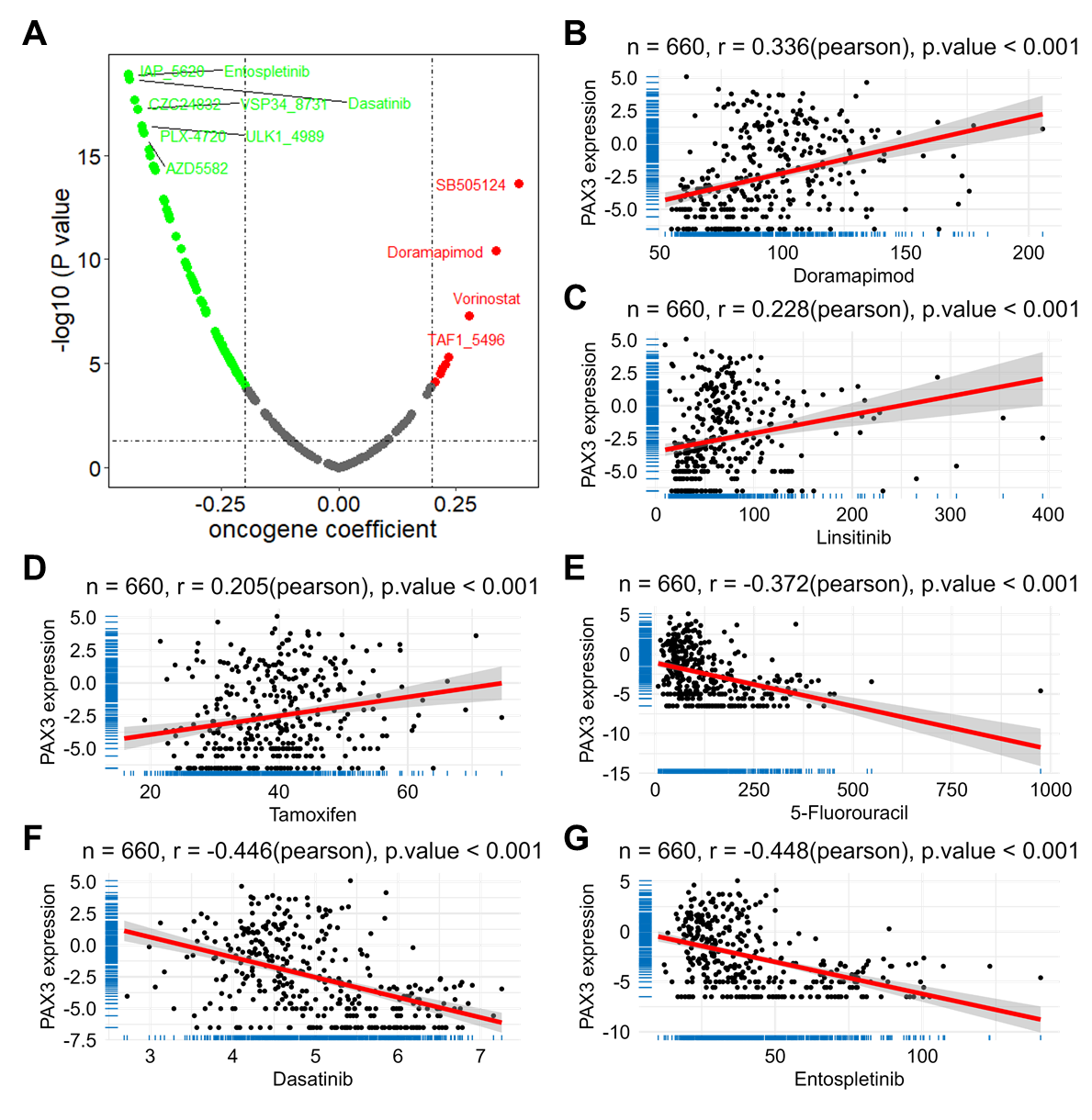


**Figure S11.** **Association of** PAX3 expression with sensitivity to multiple antitumor drugs in TCGA_LGGGBM.


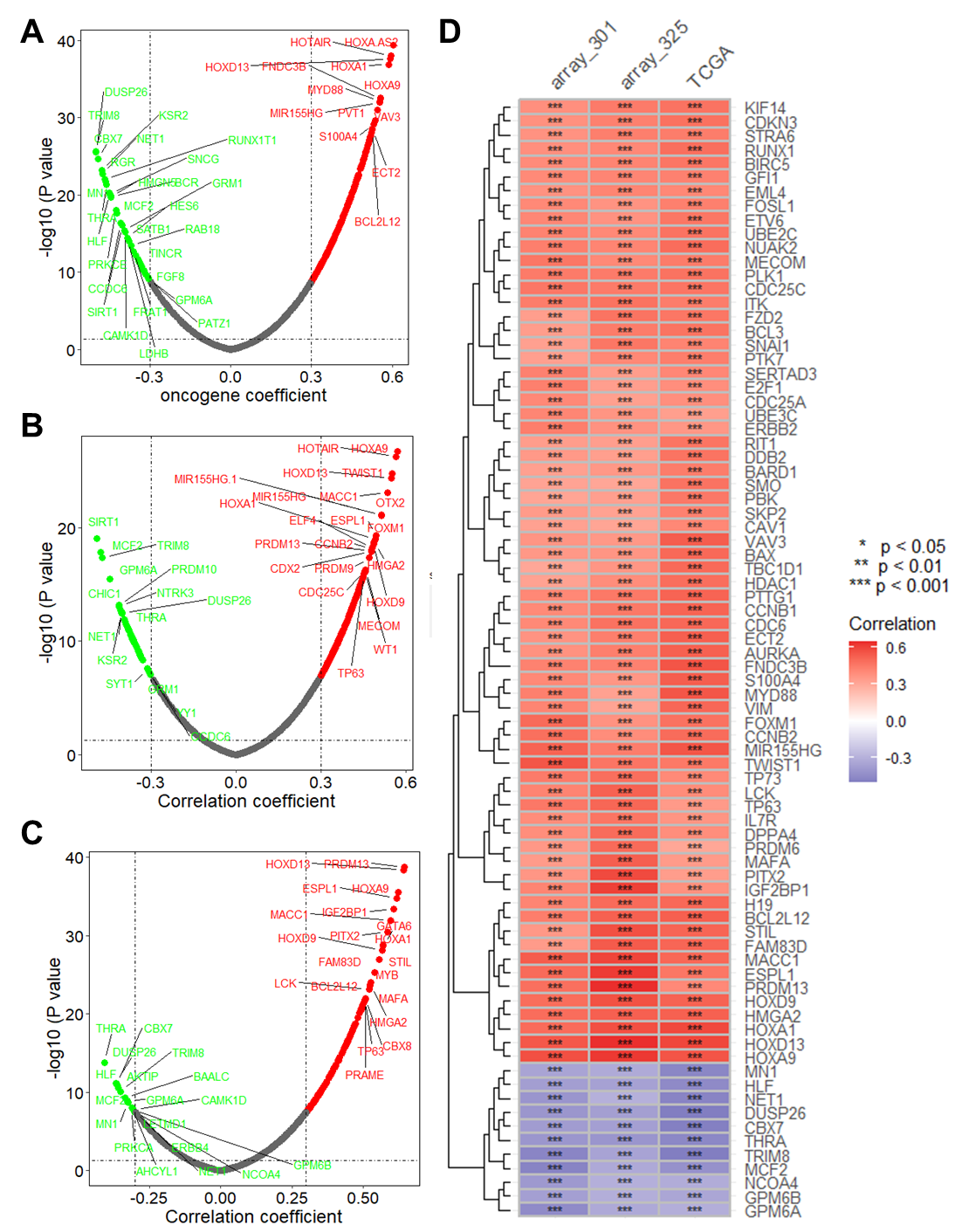


**Figure S12.** **Association of PAX3 expression with cancer gene expression.** Volcano plots show results from TCGA_LGGGBM (A), mRNA_array_301 (B), and mRNAseq_325 (C). (D) Heatmap showing overlapping PAX3-associated genes across the three datasets.
